# Small Animal Multivariate Brain Analysis (SAMBA) – a High Throughput Pipeline with a Validation Framework

**DOI:** 10.1007/s12021-018-9410-0

**Published:** 2018-12-19

**Authors:** Robert J. Anderson, James J. Cook, Natalie Delpratt, John C. Nouls, Bin Gu, James O. McNamara, Brian B. Avants, G. Allan Johnson, Alexandra Badea

**Affiliations:** 1 Center for In Vivo Microscopy, Department of Radiology, Duke University Medical Center, Durham, NC 27710 USA; 2 Department of Biomedical Engineering, Duke University Medical Center, 3302, Durham, NC 27710 USA; 3 Department of Pharmacology and Cancer Biology, Duke University Medical Center, Durham, NC 27710 USA; 4 Department of Cell Biology and Physiology, University of North Carolina, Chapel Hill, NC 27599 USA; 5 Department of Neurobiology, Duke University Medical Center, Durham, NC 27710 USA; 6 Department of Neurology, Duke University Medical Center, Durham, NC 27710 USA; 7 Biogen, Cambridge, MA 02142 USA

**Keywords:** Voxel-based analysis, MR-DTI, Pipeline, Parallel computing, Validation methods, Simulated atrophy

## Abstract

While many neuroscience questions aim to understand the human brain, much current knowledge has been gained using animal models, which replicate genetic, structural, and connectivity aspects of the human brain. While voxel-based analysis (VBA) of preclinical magnetic resonance images is widely-used, a thorough examination of the statistical robustness, stability, and error rates is hindered by high computational demands of processing large arrays, and the many parameters involved therein. Thus, workflows are often based on intuition or experience, while preclinical validation studies remain scarce. To increase throughput and reproducibility of quantitative small animal brain studies, we have developed a publicly shared, high throughput VBA pipeline in a high-performance computing environment, called SAMBA. The increased computational efficiency allowed large multidimensional arrays to be processed in 1–3 days—a task that previously took ~1 month. To quantify the variability and reliability of preclinical VBA in rodent models, we propose a validation framework consisting of morphological phantoms, and four metrics. This addresses several sources that impact VBA results, including registration and template construction strategies. We have used this framework to inform the VBA workflow parameters in a VBA study for a mouse model of epilepsy. We also present initial efforts towards standardizing small animal neuroimaging data in a similar fashion with human neuroimaging. We conclude that verifying the accuracy of VBA merits attention, and should be the focus of a broader effort within the community. The proposed framework promotes consistent quality assurance of VBA in preclinical neuroimaging, thus facilitating the creation and communication of robust results.

**Electronic supplementary material:**

The online version of this article (10.1007/s12021-018-9410-0) contains supplementary material, which is available to authorized users.

## Introduction

Computational imaging has emerged as a powerful neuroscience research tool. It has been used to identify patterns of human brain differences due to genotype, environment (Blokland et al. 2012), development (Becker et al. 2016), aging (Kremen et al. 2013), and disease (Thompson et al. 2014). The reliability of such analyses has received increased attention and scrutiny in human brain neuroimaging (Shen and Sterr 2013) (Radua et al. 2014; Michael et al. 2016) (Eklund et al. 2016). Exploring such themes in rodents provides important insight into human conditions, as phenotypes can be replicated via genetic manipulation, while environmental and other conditions can be well controlled. Indeed, murine models of neurologic diseases have played a critical role in neuroscience. It is thus crucial to develop accurate and reliable techniques specific to small animal imaging.

Our main objective is neuroanatomical phenotyping using MR histology (Johnson et al. 1993). Diffusion tensor imaging (DTI) is an attractive tool for MR histology, as it delivers multiple contrasts such as fractional anisotropy (FA) and radial diffusivity (RD) to quantify microstructural integrity (Calabrese et al. 2014). Additionally, using DTI contrasts to drive image registration can improve the resulting alignment (Badea et al. 2012). We thus need the ability to handle multiple contrasts.

Voxel-based analysis (VBA) has been established as a method for localizing and quantifying morphometric and physiological brain changes (Ashburner and Friston 2000). VBA has been used with magnetic resonance imaging (MRI), positron-emission tomography (PET), and single-photon emission computed tomography (SPECT) (Good et al. 2001; Hayasaka et al. 2004). Among these, MRI is particularly well-suited for anatomical phenotyping in small animals (Johnson et al. 2002; Nieman et al. 2005; Badea et al. 2007; Johnson et al. 2007; Borg and Chereul 2008; Badea et al. 2009; Ellegood et al. 2015). MRI-based VBA in mice has provided unique insights into conditions such as Huntington’s (Sawiak et al. 2009b) and Alzheimer’s Diseases (Johnson et al. 2010), or the effects of exercise (Biedermann et al. 2012), and the number of VBA studies continues to grow.

A major issue with VBA is the long computational time. In its most critical step, spatial normalization is realized by registering each subject to a common template. Diffeomorphic Symmetric Normalization (SyN) (Avants et al. 2008) has become the algorithm of choice for this task since Klein et al. (2009) found that it outperforms other approaches – in people, at least. A typical clinical exam features T1-, T2- or T2*-weighted scans with 1-mm isotropic voxel size, and 256x256x200 arrays, yielding about 25 MB per/scan or 75 MB per set. A DTI scan in ADNI uses a 128x128x59 array, 41 diffusion directions and 5 non diffusion weighted scans, and will produce 85 MB (ADNI 2010). In contrast, rodent brain MRI acquisitions are substantially larger (Johnson et al. 2012; Lerch et al. 2012; Calabrese et al. 2015b), and may include gradient-recalled echo (GRE) sequences at 21 μm isotropic resolution, using 1024x512x512 arrays (512 MB); and DTI protocols at 43 μm resolution, using 512x256x256 arrays. The resulting DTI parametric images, e.g. FA, are 8.5 times larger for one mouse brain relative to the human; and sum up to ~1 GB per specimen for all 7 of the standard DTI contrasts. Our multivariate analysis VBA pipeline thus needs to handle ~15 times more data than the 66 MB required for structural voxel-based morphometry in humans based in T1/T2 protocols. For such large arrays high-quality SyN registrations come with higher price tags, as a single registration of two mouse brains at 56 μm isotropic resolution can take ~100 CPU hours (VanEede et al. 2013). The best-case scenario, from a processing time perspective, would be to select one subject as the target template, requiring only (*N*-1) registrations. But this introduces a bias towards the selected specimen. To eliminate bias a better practice is to construct a study-specific, minimal deformation template (MDT) (Kochunov et al. 2001; Avants et al. 2010). Even an efficient iterative MDT strategy requires at least 3 iterations of pair-wise registrations between each MDT-contributing subject and the target template, a minimum of 3*N_*MDT*_ jobs*.* Then all subjects need to be registered to the final MDT, for a total of 4*N_*MDT*_ jobs. Consider a relatively small study consisting of 10 control (*N*_*C*_) and 10 treated (*N*_*T*_) mice, where only the controls are used to create the MDT. A total of 3*N_*C*_+(*N*_*C*_ + *N*_*T*_) = 50 jobs are necessary, or ~ 30 weeks of CPU time in the hypothetical scenario that a single CPU would be used. The numbers become more daunting as the number of subjects increases. It is therefore imperative to identify and implement efficient computational strategies for MRI-VBA.

Among possible solutions, single workstations are limited in processing power and memory. While cloud computing presents an attractive strategy, significant effort is required upfront to set up processing pipelines. Computing time, data transfer and storage are all issues to be addressed. Here we present a local cluster implementation of an automated processing that adopts parallel processing of locally stored data.

An automated processing pipeline should ensure a reproducible, tractable workflow. It also saves time by reducing human interaction, which can introduce errors, especially when many processing steps are involved. Multiple pipelines have been designed for human brain imaging, including: the FMRIB Software Library (FSL) (Smith et al. 2004; Jenkinson et al. 2012), Statistical Parameter Mapping (SPM) (Friston et al. 1994), and the LONI pipeline (Dinov et al. 2009). These however, do not translate immediately to the preclinical domain, due to difference in scale, gray/white matter distributions and contrasts, and a lissencephalic rodent brain. Image-processing pipelines for preclinical MRI neuroanatomical phenotyping have also been developed for automatic registration (Friedel et al. 2014), segmentation (Johnson et al. 2007; Badea et al. 2009; Minervini et al. 2012), label-based analysis (LBA) (Borg and Chereul 2008; Budin et al. 2013), cortical thickness (Lerch et al. 2008; Lee et al. 2011), and VBA/voxel-based morphometry (Sawiak et al. 2009b; Lerch et al. 2011; Sawiak et al. 2013; Calabrese et al. 2014). Recently Pagani et al. (2016) described a pipeline that integrates all these four functions. Little attention has however been given to evaluating computational costs, which can be drastically reduced by a high-performance computing (HPC) implementation. Given the increased array sizes, it is essential to have access to sufficient hard drive and memory (RAM) resources, which even high-end workstations may not deliver. Computing clusters provide opportunities for increasing throughput for large numbers of independent tasks (Dinov et al. 2010; Frisoni et al. 2011), as is the case for VBA. Thus, we here propose a high through put processing pipeline for small animal multivariate brain analysis: SAMBA. SAMBA takes advantage of high-performance computing (HPC), and is based on the widely used Advanced Normalization Tools package (ANTs) (Avants et al. 2009, 2014).

HPC clusters can handle massive amounts of parallel image registrations. VanEede et al. (2013) elegantly demonstrated this by completing 14½ *years*’ worth of CPU processing in approximately 2 months. However, the strength of a processing pipeline lies not just in speed. This speed enables us to produce reliable and repeatable results, and to address an unmet need for validation. Verifying VBA accuracy in preclinical studies is paramount, given the increased computational demands. Because VBA comprises multiple processing stages (e.g. spatial normalization, smoothing, and statistical analysis), even small differences in the analysis can lead to divergent conclusions (Rajagopalan and Pioro 2015). Notably, Bookstein (2001) pointed out that besides physiological sources, statistically significant effects can also arise due to missregistration. In addition, there are ongoing debates about the methodology (e.g. for registration, modulation, and statistical analysis) (Thacker 2005). These concerns can be partially addressed by visual inspection, focusing on segmented structures, or cross-validating with other modalities. Here we address the need for a quantitative substantiation of VBA studies and propose a formal validation framework.

The VBA pipeline is most sensitive to changes in the processing chain in voxel-based morphometry (VBM). Here, voxel-wise volumetric differences are calculated from the determinant of the Jacobian matrix of the deformation fields mapping each individual to a target template (Chung et al. 2001). This contrast directly encodes local volumetric information, after compensating for global changes. Access to a VBM ground truth “phantom” will enhance any quantitative validation of the system-wide performance. However, no gold standard for preclinical VBM exists. In the clinical domain Camara et al. (2006) and Karaçali and Davatzikos (2006) have simulated atrophy or hypertrophy to explore how registration affects the sensitivity of deformation recovery, an approach adapted by (VanEede et al. 2013) for the mouse brain as well. Here we propose a set of phantom images, which can be tuned to the expected deformation in a study, used to guide the selection of pipeline parameters, and employed to estimate the accuracy of the VBA results.

We show the example of phenotyping a mouse model of temporal lobe epilepsy (Lévesque and Avoli 2013). In this model, kainic acid (KA) is injected in the right basolateral amygdala, resulting in epileptic seizures, hippocampal neurodegeneration, and gliosis (Ben-Ari et al. 1980; Mouri et al. 2008; Liu et al. 2013), granule cell dispersion, and mossy fiber sprouting. Accurately recovering these changes presents a non-trivial challenge. We illustrate how VBA/VBM results span a surprisingly wide range, when varying the pipeline parameters. These choices can be informed by phantom metrics, and underscore once again the need for validation.

To address the need for valid statistical analyses, shown clearly for human fMRI (Eklund et al. 2016; Jovicich et al. 2016), but also morphometry (Hosseini et al. 2016) we incorporate several tools for parametric and nonparametric analysis in our pipeline. This, together with the automated documenting of the processing chain allow for further optimization and validation studies, and encourage “best practices” adoption (Nichols et al. 2017) for small animal imaging.

Our contributions include: 1) the development of a cluster-based VBA pipeline for multi-modal preclinical imaging; 2) an evaluation of the time efficiency gained from parallelizing the pipeline tasks; 3) a validation framework consisting of morphological phantoms and VBA-specific metrics; 4) an examination of how phantom studies inform the parameter selection, and exemplify the consequences of such selections using a mouse model of temporal lobe epilepsy. These datasets are organized in a manner that parallels recent human neuroimaging standardization efforts (Gorgolewski et al. 2016). While parallel computing is a common strategy in image processing, its adoption to VBA in small animal imaging has been limited. As the HPC implementation led to significant gains in processing efficiency, it also enabled a more thorough exploration of multiple parameter sets and evaluation metrics, both in synthetic phantoms, as well as in the case of a mouse model of epilepsy.

## Materials and Methods

### Software, Hardware, and Pipeline Overview

The VBA pipeline is scripted in Perl and built with a flexible modular structure. In addition to Advanced Normalization Tools (ANTs), software called by the pipeline include MATLAB® (The MathWorks, Inc., Natick, MA), SurfStat (Worsley et al. 2009), FSL, and the R statistical programming language (R Core Team 2015) with the Advanced Normalization Tools for R (ANTsR) package (Avants et al. 2015). Unless otherwise specified, all commands mentioned herein are from the ANTs toolkit (version/commit date: 13 October 2014 https://sourceforge.net/projects/advants/), and the *antsRegistration* command is used for all registration jobs. The pipeline runs on a Dell HPC cluster featuring the RedHat Enterprise Linux 6.7 operating system, managed via Bright Cluster Manager with Simple Linux Utility for Resource Management (SLURM) (Yoo et al. 2003) for job scheduling and resource allocation. The cluster consists of 11 nodes: a master node, and 10 CPU children nodes (Intel Xenon E5–2697), one of which offers GPU capabilities. Each child node features 16 logical cores (32 via hyper-threading) and 256-GB RAM, with a 4.2-TB hard drive system spread in redundancy across pairs of nodes, yet with the data equally accessible to all nodes.

**Fig. 1:**
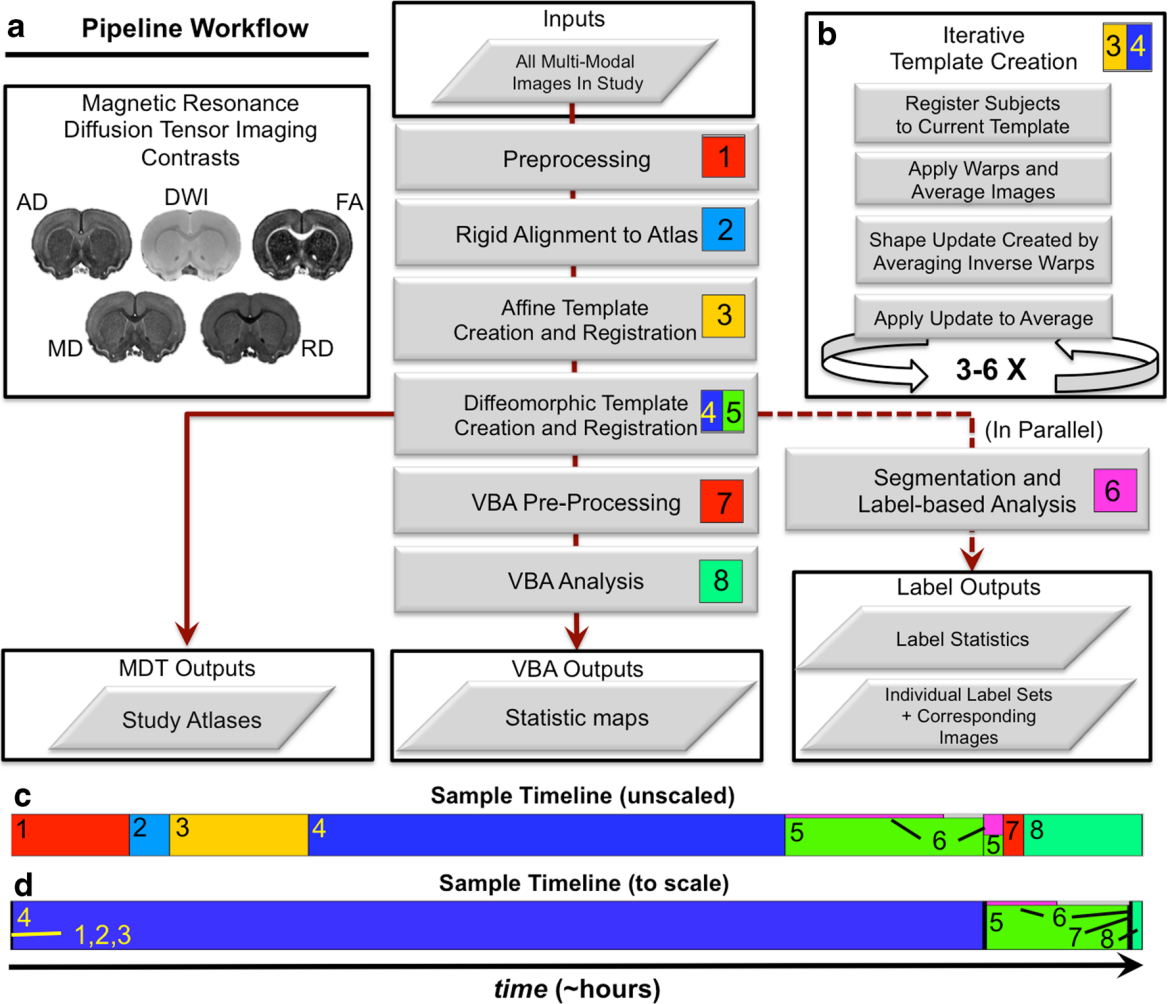
**Overview of the VBA processing pipeline.** The VBA pipeline (**a**) takes multi-modal images, such as MR-DTI contrasts (left inset), and processes them through 8 major stages. The sub-steps for iteratively creating unbiased affine and diffeomorphic targets in Stages 3 and 4 are outlined in (**b**). Study-specific atlases are generated in addition to statistical maps, while Stage 6 produces regional labels and statistics. Stages 5 and 6 run in parallel, as seen in the unscaled timeline (**c**). The total runtime is largely determined by the diffeomorphic registrations (Stages 4, 5, and 6), as illustrated by the scaled timeline (**d**)

We highlight here the key elements of the pipeline, while additional discussions of the VBA processing can be found in Supplementary Material. Figure 1a outlines the main stages of the pipeline, which handles multi-modal data, which may or may not be co-registered, e.g. MR-DTI (upper-left inset), or CT with PET respectively. Additional input required from the user is entered via a matrix of predictors, and a headfile. The matrix of predictors contains metadata of the study’s subjects (e.g. specimen ID, MR run number(s), age, gender, treatment; while the headfile is a text file including relevant processing parameters and variables. An extensive input data check is performed, and default values are assigned to missing parameters.

Stage 1 ensures the consistency and quality of the data before launching long-running jobs. Coarse spatial consistency is achieved through recentering, setting the desired orientation, and enforcing a common reference space for all images. All intra-subject acquired contrasts are rigidly co-registered, using the Mutual Information (MI) similarity metric. Other preprocessing tasks include bias field correction (if appropriate) and skull-stripping (Badea et al. 2007). The skull-stripping code included here is optimized for DWI images, and consists of histogram-based thresholding to create an initial mask. A series of morphological operations (eroding, dilating, and closing) are then performed on the mask to produce the final one that will be applied across all contrasts. This algorithm has two tunable parameters: the number of consecutive operations, and the radius of the spherical operator. Due to the critical nature of this step, the user should always pause at this step to visually ensure the quality of the masking, and adjust these parameters as necessary.

Inter-subject linear alignment is performed during Stages 2 and 3. All images are first rigidly aligned to an atlas, ideally one that provides a standard coordinate system such as Waxholm Space (“WHS”) (Johnson et al. 2010). After that, they are affinely aligned to a study-based target image. This can be one of the controls, or an unbiased average linear template created from all study subjects. The Mattes similarity metric is used for all linear registrations (Mattes et al. 2003).

In Stage 4, the MDT is iteratively created from a specified cohort of subjects. This “MDT group” is typically either the controls or all of the subjects. The key sub-stages needed to create the MDT is outlined in Fig. 1b, where both appearance and shape are optimized (Avants et al. 2010). Each cohort member is registered and warped to the current template—initially just the affine average image—and then averaged to create an intermediate template. This captures the optimal appearance of the group. Next, a “shape update” warp is created by averaging the inverse warps, and is in turn incrementally applied to the intermediate template. This diffeomorphically moves it toward the mean shape of the group, and this distance-minimized template then becomes the target for the next iteration. A true “Minimal Deformation Template” can be created in this manner with 3–10 iterations (Avants et al. 2010). This process is also ideal for creating an unbiased template in Stage 3.

Once the final template has been created, all subjects are independently re-registered to it in Stage 5. This minimizes bias towards the subjects in the MDT group. For non-linear registrations between like contrasts the cross-correlation (CC) similarity metric is used, while mutual information (MI) is used for unlike contrasts. Finally, the diffeomorphic warps from Stage 5 and the previously calculated linear transforms are used to map the original images into the MDT space.

Stage 6, Label-Based Analysis (LBA), consists of atlas-based segmentation (Gee et al. 1993). Label sets are generated via affine and diffeomorphic registration between the MDT and a labeled brain. The atlas label set is propagated to the MDT, and then to all individuals with the *antsApplyTransforms* command. A MATLAB script is used to calculate for each label its mean volume and mean values of the various contrast intensities, for each subject. Study-wide regional statistics are then computed in conjunction with the matrix of predictors. Given that diffeomorphic SyN registrations are non-linear and have many degrees of freedom, Stages 4, 5, and 6 are the computational bottlenecks of the pipeline (Fig. 1c, d).

In Stage 7 a mask derived from the MDT is eroded with a kernel of the same size as the largest smoothing kernel used, in order to avoid spurious voxels near the mask boundary. We used a 3 voxel kernel, corresponding to ~150 μm, which has worked well in our experience but may need to be tuned for different acquisitions and species. The log-Jacobian (logJac) images are calculated from the “to-MDT” warps using *CreateJacobianDeterminantImage* with the *UseGeometric* option. All contrasts are smoothed with a 3 voxel sigma Gaussian kernel, using the *SmoothImage* command.

SurfStat, ANTsR, or FSL Randomise are called to provide parametric or non-parametric voxel-based analysis in Stage 8. For parametric statistics two single-tailed t-tests are performed in opposite directions, and statistical maps are generated for the *t*-value, uncorrected *p* value, and effect size. 5000 permutations are used for nonparametric statistics. Subsequently the multiple-comparison correction is done using False-Discovery Rate (FDR) (Genovese et al. 2002) to produce q-values.

### Animals and Specimen Preparation

Animal procedures were approved by the Duke University Institutional Animal Care and Use Committee. To model epileptogenesis, a 33G cannula (Plastics One) was stereotactically inserted into the right basolateral amygdala of anesthetized C57BL/6 mice (*n* = 10), and KA (0.3 μg in 0.5 μl phosphate-buffered saline [PBS]) was infused at a rate of 0.11 μl/min (Liu et al. 2013). A cohort of 10 control animals was infused similarly with PBS. Twelve weeks following the infusion, the brain specimens were prepared for scanning, as described in (Johnson 2000; Johnson et al. 2002; Johnson et al. 2007). After being anesthetized to a surgical plane, mice were perfused through the left ventricle with outflow from the right atrium. The blood was flushed out with 0.9% saline at a rate of 8 ml/min, for 5 min. Fixation was done via perfusion with a 10% solution of neutral buffered formalin phosphate containing 10% (50 mM) Gadoteridol (ProHance, Bracco Diagnostics Inc., Cranbury, NJ), at 8 ml/min for 5 min. The heads were removed and soaked in 10% formalin buffer for 24 h, before being transferred to a 0.01 M PBS solution containing 0.5% (2.5 mM) Gadoteridol at 4 °C for 5–7 days. This reduced the spin lattice relaxation time (T1) of the tissue to ~100 ms. Extraneous tissue was removed, and specimens were placed in MRI-compatible tubes, immersed in perfluoro polyether (Galden Pro, Solvay, NJ) for susceptibility matching and to prevent dehydration.

### Image Acquisition and Post-Processing

All specimens were scanned on a 7-Tesla small animal imaging system equipped with an Agilent VnmrJ 4 console. A custom silver solenoid coil (*d* = 13 mm) was used for RF transmission and reception. MR-DTI images were acquired using a 3D diffusion-weighted spin-echo sequence with repetition time (TR) = 100 ms, echo time (TE) = 14 ms, and b-value = 1600 s/mm^2^. The image array size was 400x200x160, over a 20.0 × 10.0 × 8.0 mm field of view, producing 50 μm isotropic image resolution. The diffusion sampling protocol included 6 diffusion directions (Jiang and Johnson 2010) and 1 non-diffusion-weighted (b0) measurement. Total acquisition time was 7 h. After registering all individual DWI images (each sensitized to a different diffusion direction) to the b0 image with an affine transform we used the Diffusion Toolkit (Wang et al. 2007) to estimate the diffusion tensor and calculate the mean diffusion-weighted image (DWI), axial diffusivity (AD), fractional anisotropy (FA), mean diffusivity (MD), radial diffusivity (RD), and apparent diffusion coefficient (ADC). The DTI parametric images were used as the input for the VBA pipeline.

### VBA Processing

To examine the effect of template construction we ran the VBA pipeline for two scenarios (controls vs. phantoms; and control vs. KA-injected animals), using 12 different registration parameters sets, and two template generation strategies, for a total of 48 times. The first strategy used only the control animals to construct the MDT (denoted as “C”). In the second strategy all the animals contributed to the MDT (“A”), similarly to (Avants et al. 2010). For a given set of registration parameters, both the phantom and KA runs used the same MDT for “C”, while for “A” a new MDT needed to be generated with each pipeline run.

We ran the first three stages of the pipeline only once, because it was not until Stage 4 that any parameters were varied. For these common stages, we ran the skull-stripping algorithm with DWI images, using the default parameters of 5 morphological operations with a radius of 2 voxels. The quality of the resultant masks was visually inspected and ensured before proceeding, a recommended best practice. We used the Waxholm Space mouse brain atlas (Johnson et al. 2010; Calabrese et al. 2015b) to provide the orientation for rigid registration. A native image from the study, padded along y (the left-right axis) with 12 voxels, defined the reference space. Thus, the final array size was 400x212x160 with 50 μm isotropic resolution. One control subject was arbitrarily selected as the target for affine registration. For both the rigid and affine stages, DWI images were registered with a gradient step of 0.1 voxels, using the Mattes similarity metric (32 bins, 1e-8 convergence threshold, 20-iteration convergence window). Registration was constrained to two down-sampled levels of 6x and 4x, with a maximum of 3000 iterations, with smoothing sigmas of 4 and 2 voxels, respectively. *Histogram matching* and *estimate learning rate once* options were used.

The pipeline was run multiple times, diverging at Stages 4 and 5. Here we varied the three SyN-specific parameters required by *antsRegistration*: the gradient step size (referred to here as the singular “SyN” parameter), a regularization parameter for the velocity/“*update*” field (“RegU”), and a regularization parameter for the *total* warp field (“RegT”). Previous work in our lab involving smaller deformations in an Alzheimer’s disease mouse model suggested values of (0.5, 1, 0.5) to provide a balance between quality and computation time (Badea et al. 2007, 2012), and we use these as a starting point. We chose SyN values of 0.1, 0.25, and 0.5 voxels. The smaller SyNs allowed us to examine the trade-off between higher-quality results and increased run time. RegU assumed larger values of 3 and 5 which are more appropriate for recovering large deformations in a reasonable timeframe. Finally, RegT took on values of 0 and 0.5 voxels, the former at the suggestion of the creator of the ANTs software package. Thus the parameter space of *MDT*(*SyN*, *RegU*, *RegT*) was 2x3x2x2 = 24 permutations. In the absence of metrics to guide our selection, our “best-guess” was *C*(0.25, 3, 0,5).

For the MDT creation in Stage 4 we used iterated the process 6 times, the first 3 of which were decreasingly down-sampled. FA images were used to drive all diffeomorphic registrations via the cross correlation (CC) similarity metric with a 4-voxel radius and dense sampling. The convergence threshold and window were the same as in previous stages. We used 4 sampling levels, 8x, 4x, 2x, and 1x, with a maximum of 4000 iterations each; with smoothing sigmas of 4, 2, 1, and 0 voxels, respectively. A 3-voxel radius was used for smoothing the images, before voxel-wise statistical analysis.

### Temporal Performance of the Pipeline

To examine computational efficiency we simulated the runtime of the *C*(0.25,3,0.5) KA analysis when using a high-end workstation and the cluster with 1–6 nodes (*n*_*nodes*_). We compared the runtimes for the 24 registration parameters sets when using 4 nodes. In practice, the desired number of multi-threaded registration jobs were assigned to a given node by requesting the appropriate integer fraction amount of memory when calling Slurm, while allowing them to share the cores on that node.

We evaluated: 1) the real (“wall-clock”) time for each cluster job; 2) its corresponding total CPU time (processing time of the workload normalized to one processor); 3) a conversion factor relating the two; and 4) the distribution of jobs across *n*_*nodes*_ during a given Stage. Slurm’s *sacct* command provided the first two quantities via its *CPUTime* and *TotalCPU* fields. From this, we estimated the *CPUTime*/*TotalCPU* conversion factor to be 0.0325 ± 6.6e-04, very close to the theoretical limit of 1/32 (0.0312) for 16 hyper-threaded cores. Lastly, given that jobs are to be distributed evenly across nodes, the lists of jobs for each node were easily determined. Each node’s workload was calculated by summing the *TotalCPU* of all its jobs, and converted to real time (*CPUTime*). A Stage’s runtime was taken to be the longest of these *CPUTimes*. We only considered the jobs from Stages 4 and 5, since these rate-limiting steps serve as an excellent proxy for the temporal performance of the entire pipeline. The combined Stage 4 and Stage 5 runtimes, sorted by constant parameter groups, were log10 transformed to improve normality and to illustrate relative changes in compute efficiency, before performing paired *t*-tests. Resulting effect sizes are thus reported as runtime multipliers.

To compare temporal performance between a workstation and the cluster we calculated a conversion factor based on the average iteration time over the same 3 randomly selected SyN registration jobs. The 3 jobs were run in parallel on a single cluster node, and in serial on our most powerful workstation (12 cores [24 hyper-threaded] × 2 Intel Xenon E5–2650). We chose serial processing on the workstation because the ability to run parallel jobs is RAM-limited.

### Manual Labels and Dice Coefficients

An atlas based segmentation using a novel symmetrized atlas featuring 166 regions on each side, and having as initial point the parcellations described in (Calabrese et al. 2015b) provided label sets for 5 KA brains. The automated labels were generated using *C*(0.5,3,1) and provided the starting point for manual corrections. Four regions, left/right hippocampus (Hc) and left/right caudate-putamen/striatum (CPu), were then manually delineated. The same person (RJA) performed all segmentations using Avizo (FEI, Burlington, MA), guided by multiple contrasts (AD, MD, and RD).

Once each pipeline run completed, the resulting label set was used to calculate Dice coefficients, the “silver standard” for evaluating spatial registration (Avants et al. 2011). These were generated via the ANTs *LabelOverlapMeasures* command. Ipsilateral to the injections, the right Hc Dice values characterize how well atrophy was recovered. The left CPu functioned as a control, as it was minimally impacted by the KA. The left Hc and right CPu were pseudo-controls, featuring structural correlation with the right Hc. An in-depth analysis of the Dice coefficients is in the Supplemental Material. There, the values from the same subject were paired, such that they had 3 of the 4 registration/MDT parameters in common. This kept all variables constant except for one, and its effect could be measured using paired *t*-test across all combinations of constant parameters and subjects. For SyN, three separate *t*-tests were performed, (0.1 > 0.25), (0.1 > 0.5), and (0.25 > 0.5). For each t-test, *n*_*pairs*_ = 60 (24 parameter sets*5 specimens/2 groups), except for the SyN comparisons, where *n*_*pairs*_ = 40 (24 *5 /3).

### The Validation Framework

**Fig. 2:**
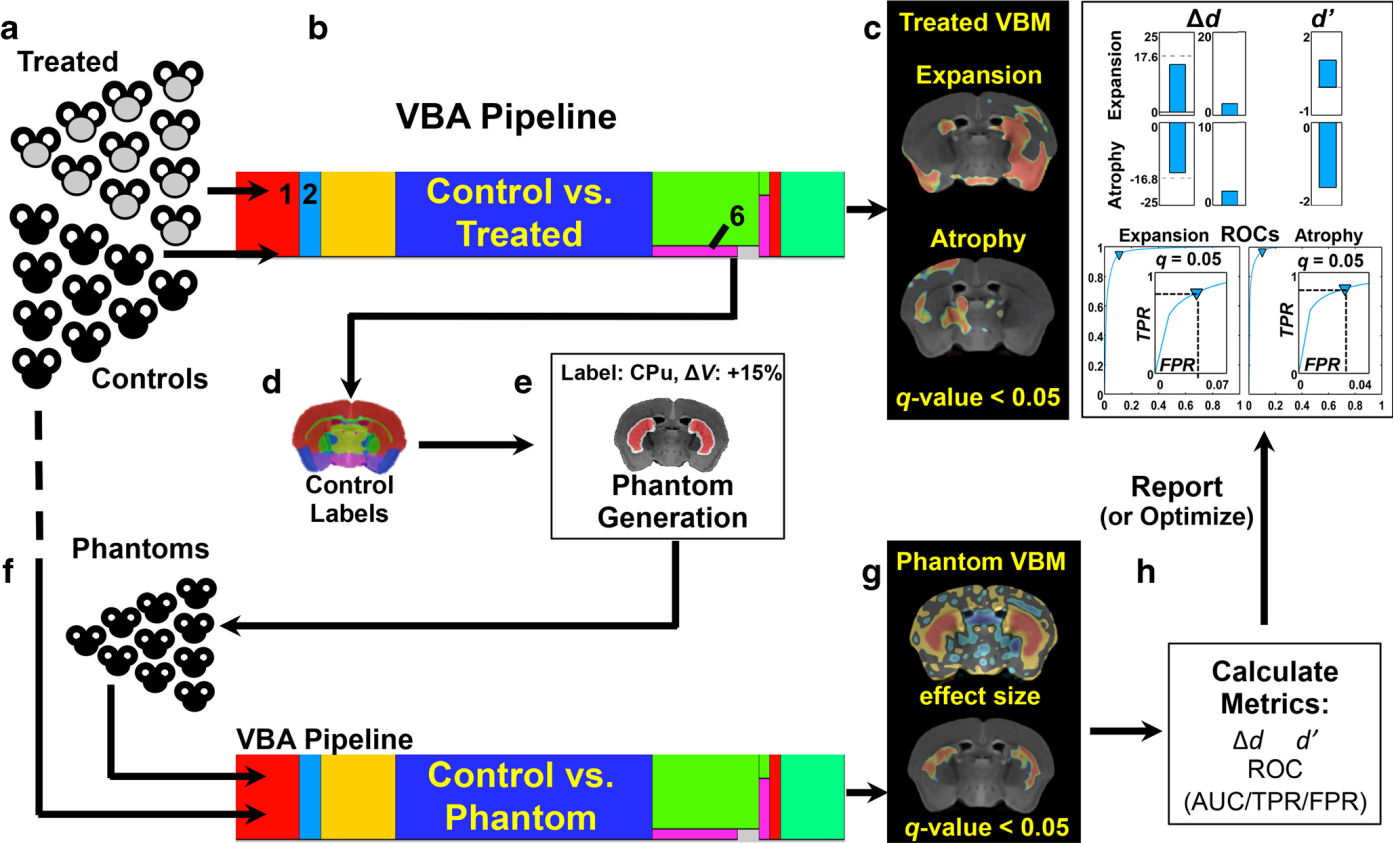
**Overview of the VBA validation framework.** Control and treated images (**a**) are fed into the VBA pipeline, initialized using our best-guess SyN parameters (**b**), ultimately producing statistical results (**c**). During this process, once automated label sets are available for the control images during stage 6 (**d**), they become input for phantom creation (**e**). The user can specify how much atrophy or hypertrophy to induce in the structures of their choice. The pipeline is reinitialized, this time with the control and phantom images (**f**). The results of the phantom VBA (**g**) are used for calculating several metrics (**h**), reported alongside the regular VBA results, or used to optimize the SyN or smoothing parameters

We propose a framework for evaluating VBA workflows in the small animal brain (Fig. 2). This is based on simulated morphological changes, and quantifying their subsequent recovery. There are two primary components: morphological phantom creation, and metric calculation based on VBM.

#### Generation of Morphological Phantom Data

Our primary goal in validating the pipeline was to recover the atrophy or hypertrophy induced in our phantoms. Specifically, we induced hypertrophy in the left CPu and atrophy in the right Hc. An asymmetric approach helps better isolate the opposing morphometric changes; and allows for testing whether any software in the pipeline reverses L-R axis. Creating phantoms with atrophic Hc suited our evaluation purposes since we expected hippocampal atrophy in the KA-injected mice. Simulating atrophy in Hc provided insight into the expected performance for the actual KA group.

**Fig. 3:**
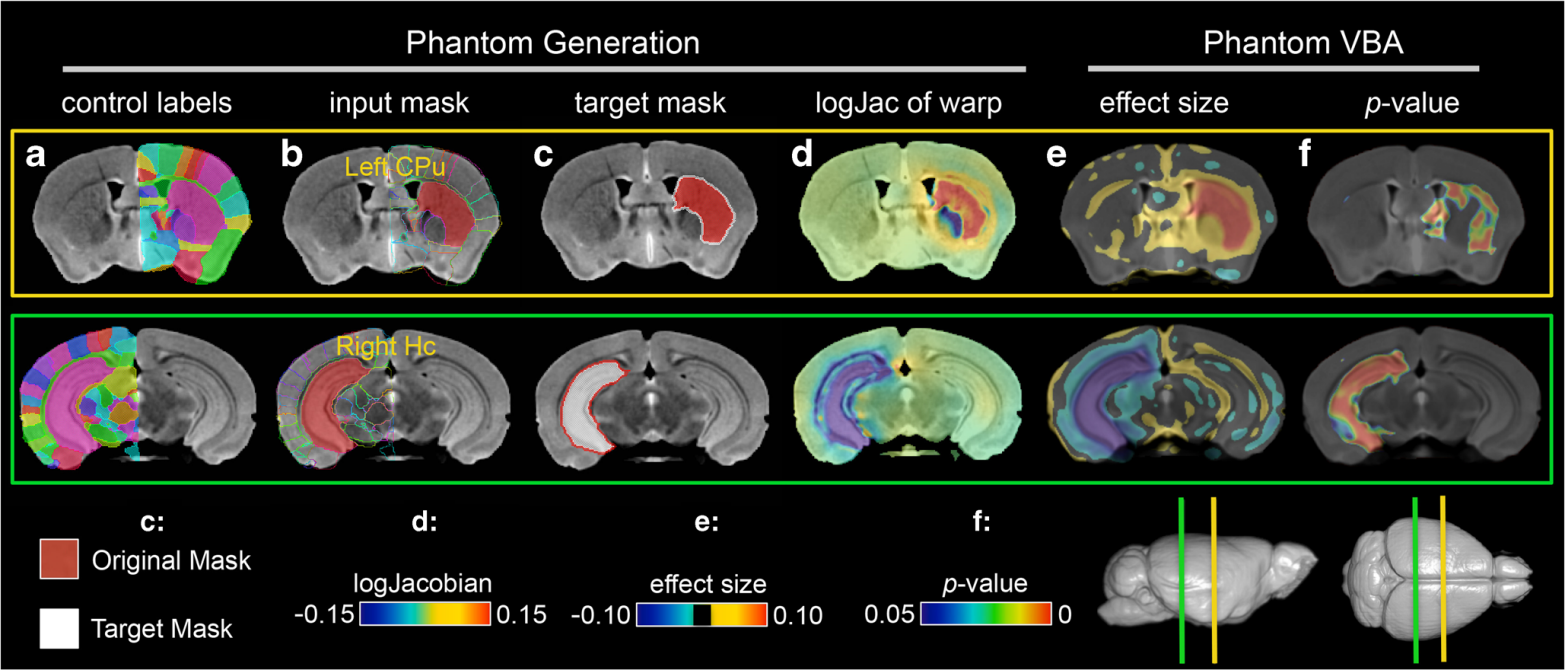
**Inducing asymmetric morphological changes in control images generates a set of VBM phantoms.** The label sets (**a**) of control images generated during Stage 6 were used to create input masks (**b**) for the left caudate-putamen (CPu, top) and the right hippocampus (Hc, bottom). Localized morphological changes are created by dilating (CPu) and eroding (Hc) the input masks to create target masks (**c**). The original masks are diffeomorphically registered to the target masks, producing a warp which relates the original image to the phantom image. The natural log of the Jacobian determinant of the warp (**d**) reflects the regional volume changes. There is excellent spatial correspondence between the inputs and outputs, with a nominal amount of leakage of the effect size (**e**) outside the mask regions. The leakage decreases substantially when *p* < 0.05 (**f**)

We generated phantom images (Fig. 3), with modest target volumetric changes of ~ ± 14%. We started with a set of control images and their corresponding label sets (Fig. 3a), the latter of which had been automatically produced in Stage 6 with the “best-guess” parameters, *C*(0.25,3,0.5). From these, we used MATLAB to extract subject-specific binary masks corresponding to the left CPu (**3.B,** top) and right Hc (**3.B**, bottom). The *imdilate* and *imerode* MATLAB commands were used to alter the regions, until they approached the target volume. Here, the CPu mask was dilated and Hc mask was eroded each by one voxel (**3.C**) to reach the ~ ± 14% targets. The two modified masks were then recombined into a single target mask. The original masks were merged and diffeomorphically registered to the target mask with registration parameters (0.5,3,1). Since the phantom generation is based on a simple model of expansion/contraction of a volume, it was appropriate to use a *RegT* of 1 (as opposed to 0 or 0.5) to better constrain the resulting warp. The MeanSquares image similarity metric was used with full sampling. To illustrate the voxel-wise volumetric change induced across each structure, Fig. 3d shows the log-Jacobian for the target-to-original warp. Values less than zero represent atrophy, and greater than zero, hypertrophy. The phantom images for each subject were produced by applying the resulting warp to all contrast images with *antsApplyTransforms* using linear interpolation (**3.E**). Creating phantoms for 10 subjects took ~14 min for 400x212x160 images, when running in parallel on the cluster. We measured the induced volumetric changes through the mean of the Jacobian across the original masks. The average percent change across all subjects was used as the “target” for evaluating performance. Specifically, the one-voxel dilation of the CPu corresponded to a 13.6 ± 0.8% change in volume (0.128 in terms of log-Jacobian), while the one-voxel erosion produced a −13.6 ± 1.7% change in the Hc (−0.146 log-Jacobian).

Figure 3 also illustrates how well VBA recovers the induced morphological changes in phantom images. We present here the results for our best-guess SyN parameters, *C*(0.25, 3, 0.5). While the effect size (Fig. 3e) exhibits what might be considered noise, the majority of this is eliminated when considering voxels with *p* values below 0.05 (**3.F**). As desired, the clusters of statistical significance are largely confined to the input masks.

#### Evaluation Metrics for Phantom Analysis

We propose four quantitative metrics for evaluating performance of the VBA pipeline: the *distance from target*, the sensitivity index, and the Area Under the Curve (AUC) and True Positive Rate (TPR) at *p = 0.05* obtained from Receiver Operating Characteristic (ROC) plots. These respectively quantify the accuracy (in sign and magnitude) of the reported effects, the spatial precision, and the expected tradeoff between true and false positives at various statistical thresholds. Although not scalar metrics, ROC plots are included as well.

The *distance from target* is defined as the absolute distance between the simulated volumetric change and the value implied by the effect size, measured in percentage. The effect size, in the native units of the logJacobian, is averaged across each structure in which we have induced change. The implied volume change in percent is: Δ*V*_*implied*_ = ((*exp*(*effect*_*structure*_)-1) × 100%. Consequently, the distance from target is: Δ*d* = |Δ*V*_*simulated*_ - Δ*V*_*implied*_|. Only the absolute value |Δ*d*|, is considered here, although in some cases it may be of interest if the volumetric change is being overestimated. It is desirable to minimize this total distance as it indicates higher accuracy, and thus we plot it with the *y*-axis inverted.

To quantify the localization of the induced effects we used a sensitivity index, *d’* (“d-prime”) (Green and Swets 1966), and looked for “effect leakage”: nearby falsely significant effects primarily arising from bias related to the model priors employed during spatial normalization. We treat the effect size as a signal and the sensitivity index is: *d’* = (μ_S_ – μ_N_)/√(σ^2^_S_ + σ^2^_N_). Here, μ_S_ and σ_S_ are the mean and standard deviation of the signal, and μ_N_ and σ_N_ of the noise, with d-prime indicating how readily a signal can be detected. With perfect registration, the effect size within an altered structure (“signal”) should be easily distinguished from effect size immediately outside of the structure (“noise”). We create binary masks for the noise, referred to as the leakage regions, by dilating the masks of the altered structures by two voxels, then removing the original generating structure and any voxels that belong to neighboring structures that have also been altered. Likewise, an inner shell for each structure is created to approximately match the volume of the corresponding adjacent leakage region. The effect size within this region is considered rather than the entire structure. To estimate d-prime we measured the distribution of the effect size within the inner shell and within its leakage region.

Determining whether a voxel is significant is a binary classification task based on a threshold, and can be characterized by an ROC. We constructed ROCs based on *p-* and *q*-values. Ideally, all voxels within an altered structure would be significant (True Positives), but none outside (False Positives). For a given threshold, the TPR is the fraction of significant voxels within the structure. The False Positive Rate (FPR) is the amount within the brain, but outside the structure. An ROC is constructed by plotting the FPR along the *x*-axis and the TPR along the *y*-axis; and the AUC is calculated by approximating the area with finite trapezoids.

Each metric was calculated for the 24 parameter sets for the right Hc (atrophy) and the left CPu (hypertrophy), and then are group−/pair-wise sorted in the same fashion as the Dice coefficients by varying 1 of the 4 parameters at a time. Similarly, paired *t*-tests were performed across the constant parameter groupings for these 4 phantom metrics and the average Dice coefficients, and the *p* values and median effect sizes were recorded. Based on these pairwise comparisons, we selected two scenarios for side-by-side comparison of the KA VBA results, in which the impact of varying one parameter at a time—RegT and SyN in this case—was visually apparent. More scenarios, including the variation of RegU and MDT group, are included in the Supplemental Material, together with the results for the SyN(0.1 > 0.5) and SyN(0.25 > 0.5*) t*-tests*,* and scatter plots examining the correlation between the Dice coefficients and the phantom metrics.

To produce an average phantom ranking the 24 parameter sets were ranked according to each metric, and ranks were averaged. The average phantom and Dice ranking, as well as the runtimes were computed to integrate all metrics available. The KA VBA results corresponding to the extremes and the median of the ranked results were compared, to illustrate the variation in unguided VBA.

## Results

To address preclinical imaging needs, we have developed a cluster-based VBA pipeline for small animal multivariate brain analysis, SAMBA, which we have tested extensively using DTI parametric images. We propose a VBA validation framework consisting of morphological phantoms and VBA-specific metrics. We have used SAMBA in a thorough evaluation of time efficiency gained from parallel processing, and applied it to a model of epilepsy, illustrating the wide effects of parameter choices on VBA, and how phantoms can inform a parameter’s selection.

### Temporal Performance

**Fig. 4:**
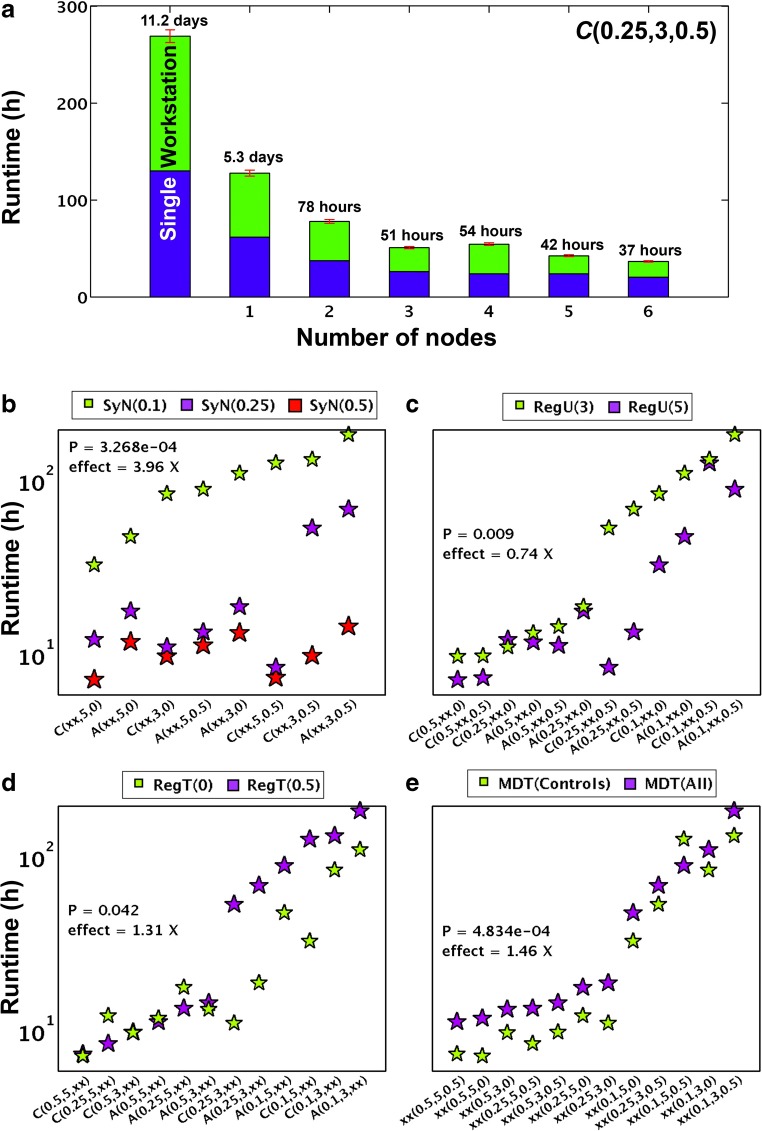
**VBA pipeline runtimes and their relationships with registration parameters and MDT construction strategy.** The workloads of Stages 4 (blue) and 5 (green) for the best-guess (0.25,3,0.5) KA run indicate that a speedup >7 can be achieved using 6 cluster nodes (**a**). Log_10_ of the runtimes are plotted for the comparison of the SyN parameters (**b**). The largest impact (~ 4X) comes from using a SyN parameter of 0.1 instead of 0.25 (**b**). Also shown are the comparisons for parameters: RegU (**c**), RegT (**d**), and MDT group (**e**)

A major advantage of HPC is the increased throughput. Figure 4a shows the runtimes for Stages 4 and 5 using *C*(0.25,3,0.5) for a single workstation, as well as 1–6 cluster nodes. Compared to serial job scheduling on a workstation with a similar processor (first bar), we noted a speedup of ~2.11 by moving to the cluster. The ability to run parallel jobs with high memory requirements is a clear advantage of HPC, even if only using one node. Each additional node increases this factor by ~0.8. Adding nodes decreased the total runtime, approaching the lower limit of 1/n. Using 6 nodes, the VBA time decreased from ~1.5 weeks to ~1.5 days, an 86% increase in efficiency.

The VBA pipeline runtimes ranged from 7.3 h for *C*(0.5,5,0) to 187 h (7.8 days) for *A*(0.1,3,0.5). The largest effect of changing any one parameter was attributed to SyN (Fig. 4b). SyN(0.1) runs typically took 4x longer than SyN(0.25). Using RegU(5) instead of RegU(3) resulted in a ~25% reduction in runtime (**4.C**). Similarly, a modest difference (~30%) came from choosing RegT(0.5) (**4.D**). The ~45% increase in runtime when creating an MDT from all the subjects was consistent across comparisons (**4.E**). In conclusion, the time penalty was high when SyN was small, or even when SyN was modest and RegU was small. This suggests that better performing parameter groups require longer runtimes, and a runtime penalty must be taken into account when selecting parameters.

### Evaluation of Processing Parameters Via Dice Coefficents and Phantom VBA

Kainic acid Dice values in the Hc (both left and right) ranged from 86.66% to 95.49%, with a mean of 92.58 ± 1.35%. Across the CPu, the Dice ranged from 88.89% to 95.20%, with a mean of 93.46 ± 1.26%. Subject-wise paired *t*-tests are tabulated and visualized in **Supplemental Table****S1** and **Fig.****S1**, respectively. Results of the comprehensive paired *t*-tests are shared in Table 1, and illustrate the impact of each parameter choice in terms of both Dice coefficients and phantom metrics.

**Table 1:** The *p* values and effect sizes of the pairwise *t*-tests of SyN, RegU, RegT, and MDT, for Dice coefficients and phantom metrics

Parameter	*Dice*	|Δd|			*d’*		*AUCx100*		*TPR@p = 0.05*	
Comparison	*Hc*	*CPu*	*Hc*	*CPu*	*Hc*	*CPu*	*Hc*	*CPu*	*Hc*	*CPu*
SyN: 0.1 > 0.25										
Effect size:	0.42%	0.27%	0.18%	0.17%	**0.143	**0.168	**0.09	**0.35	**9.5%	**24.5%
*p* value:	0.069	0.251	0.028	0.071	0.002	0.002	2.3e-04	1.4e-04	1.3e-4	7.3e-04
SyN: 0.1 > 0.5										
Effect size:	0.52%	0.37%	0.30%	0.05%	**0.148	**0.180	**0.13	**0.46	**11.3%	**31.5%
*p* value:	0.049	0.064	0.008	0.366	0.003	0.002	2.1e-05	8.8e-05	8.2e-05	7.6e-04
SyN: 0.25 > 0.5										
Effect size:	0.16%	**0.16%	0.10%	−0.16%	0.016	0.031	**0.03	0.08	**2.3%	4.1%
*p* value:	0.110	6.3e-04	0.212	0.102	0.189	0.016	0.0025	0.048	0.002	0.015
RegU: 5 > 3										
Effect size:	−0.01%	−0.08%	**-0.14%	−0.21%	**-0.085	**−0.070	**−0.07	**−0.19	**−4.8%	**−0.2%
*p* value:	0.418	0.688	4.4e-04	0.019	1.3e-05	8.2e-05	1.5e-04	2.3e-04	1.9e-04	3.6e-04
RegT: 0.5 > 0										
Effect size:	**0.93%	**0.82%	0.1%	**0.31%	**0.159	**0.142	**0.22	**0.85	**24.0%	**39.8%
*p* value:	8.3e-04	3.1e-04	0.158	5.8e-04	9.1e-07	1.9e-05	5.3e-08	1.3e-09	3.1e-09	1.3e-09
MDT: All>Ctrl										
Effect size:	**0.83%	0.03%	**0.14%	−0.05%	0.013	**−0.035	**0.02	−0.07	**−1.4%	−2.0%
*p* value:	8.5e-06	0.227	4.2e-04	0.061	0.020	3.3e-05	0.002	0.011	0.003	0.005

**indicates effect sizes with *p* value <0.005

Overall, the RegT and SyN produced the largest effects in both Dice and phantom metrics. RegT paired *t*-tests featured the smallest *p* values and, in AUC and TPR, effect sizes ~2x greater than the closest values produced by SyN. RegU had a smaller but significant effect per the phantom metrics, which was not captured by the Dice coefficients. The choice of MDT group had a sizable effect per the Dice coefficients, particularly in the atrophied right KA hippocampi, while the phantom metrics incorrectly did not capture this effect. The underlying data from Table 1 was also used to determine which phantom metric best correlated to the Dice (see Supplemental Fig. S7). AUC was most tightly correlated to the Dice with *R* = 0.708, *p* = 1.076e-4 (atrophy) and *R* = 0.836, *p* = 3.75e-7 (hypertrophy).

**Fig. 5:**
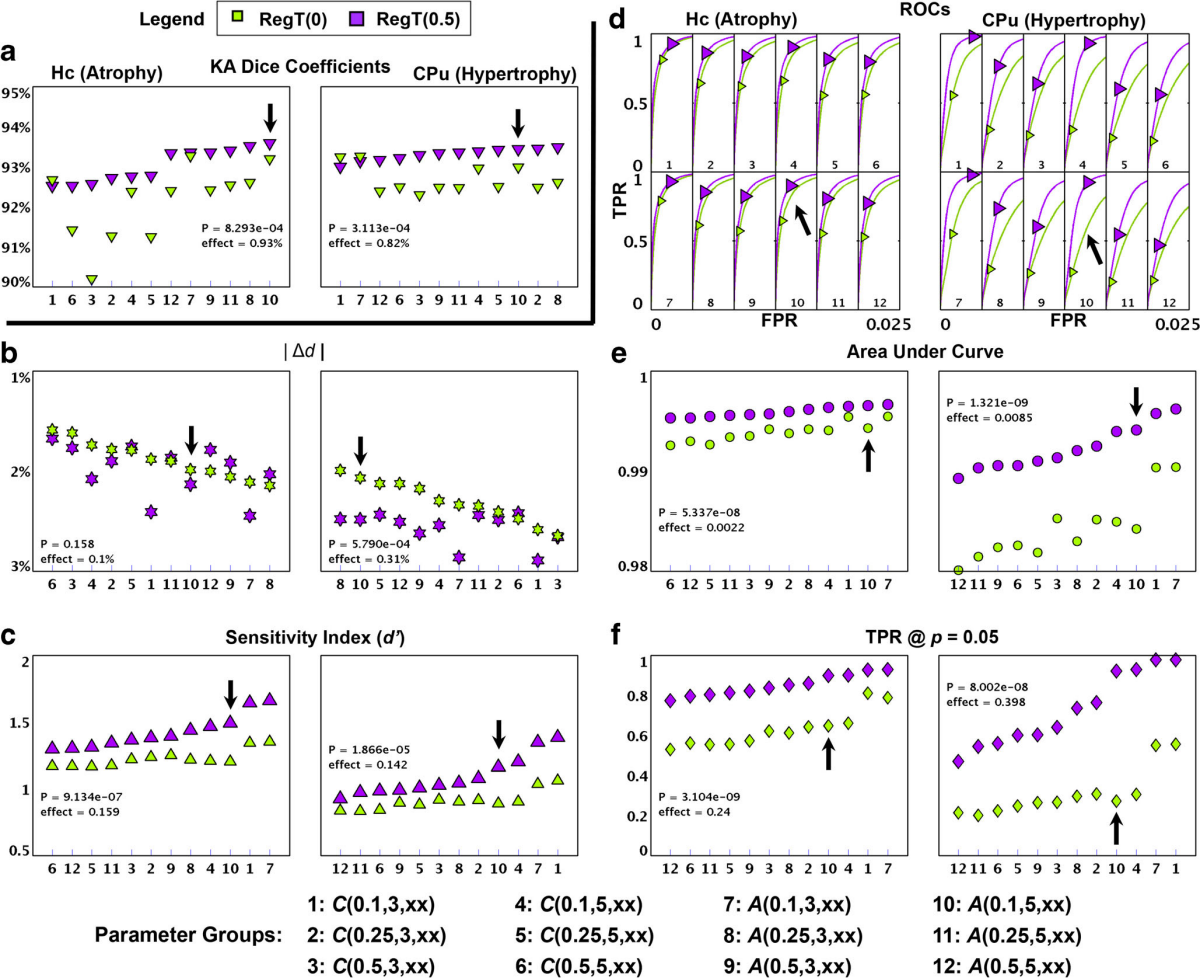
**The Dice and phantom metrics reveal the significant impact of RegT.** The left panel of each subplot corresponds to atrophy in the Right Hc, while the right panel characterizes hypertrophy in the Right CPu (Dice coefficients) and Left CPu (phantom metrics). The *x*-axis has been sorted according to the parameter value with the best mean value of that particular metric. Each group or pair with a common location on the *x*-axis represents pipeline runs featuring identical registration/MDT parameters, except for the varying parameter of interest (denoted by “xx”). The first letter identifies the MDT cohort—“C” for controls and “A” for all subjects—while in parentheses are the registration parameters (SyN, RegU, RegT). For both the Dice coefficients of the kainic acid injected mice (a), and the phantom VBA metrics (b-f), increasing RegT from 0 (green) to 0.5 voxels (purple) produced significant improvements. However the absolute distance from target |Δ*d*| was an exception (b). By this metric, RegT(0.5) was less likely to recover the induced deformations. The same trend of |Δ*d*| being an outlier amongst other metrics was observed for other parameter comparisons as well. The arrows highlight Group 10, *A*(0.1,5,xx), chosen for the KA VBA comparison in Fig. 6

**Fig. 6:**
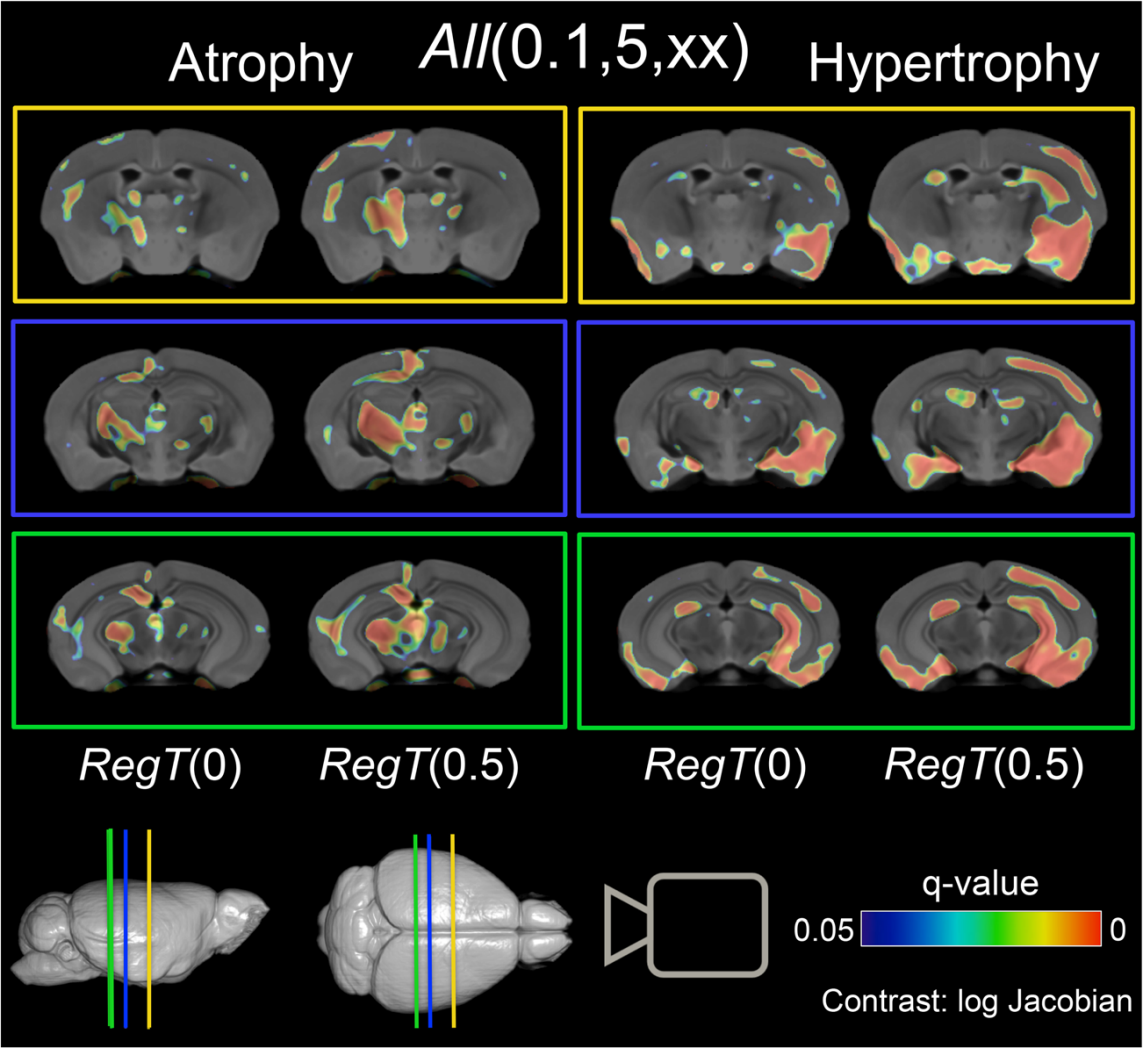
**The impact of RegT on the kainic acid VBA results, illustrated by corrected*q*-maps.** The parameter group *A*(0.1,5,xx) demonstrated very strong effects when varying the RegT parameter. Atrophy (left) and hypertrophy (right) are mapped for three coronal slices. Both atrophy and hypertrophy feature larger clusters for RegT(0.5). The detected hypertrophy is greatly diminished in the contralateral cortex, caudate putamen, hippocampus, and amygdala

Figure 5 illustrates the impact of varying RegT as reflected by the performance metrics (see also the Supplemental Figs. S2, S3, and **S5** for the effects of varying SyN, RegU, and the MDT group). The automated labels of the 24 KA VBA runs are used for calculating average Dice coefficients (Fig. 5a). The metrics based on the 24 phantom VBA runs include: absolute distance from target (**5.B**), sensitivity index (**5.C**), the Receiver Operating Characteristic (ROC) plot (**5.D**), ROC Area Under Curve (**5.E**), and ROC TPR at *p* = 0.05 (**5.F**). Compared to other parameters RegT(0.5) produced the largest significant effects on Dice coefficients (**5.A**). An unexpected trend emerged in |Δ*d*| (**5.B**), in which the effects due to changing a given parameter were either highly variable or in favor of values that perform more poorly per other phantom metrics. RegT induced significant variation in all phantom metrics (apart from |Δ*d*|). This is evident in *d’* (**5.C**), AUC (**5.E**), and TPR (**5.F**). We chose Parameter Group 10, (*A*(0.1,5,xx), indicated by the arrows in Fig. 5) because of its large impact on most metrics, and assessed the corresponding KA results (Fig. 6). Both atrophy and hypertrophy were more expansive if using RegT(0.5) relative to RegT(0), notably in the contralateral cortex, caudate putamen, hippocampus, and amygdala. Differences were large enough that varying RegT could lead to divergent conclusions, suggesting that using non-zero RegTs will provide stability in the results.

**Fig. 7:**
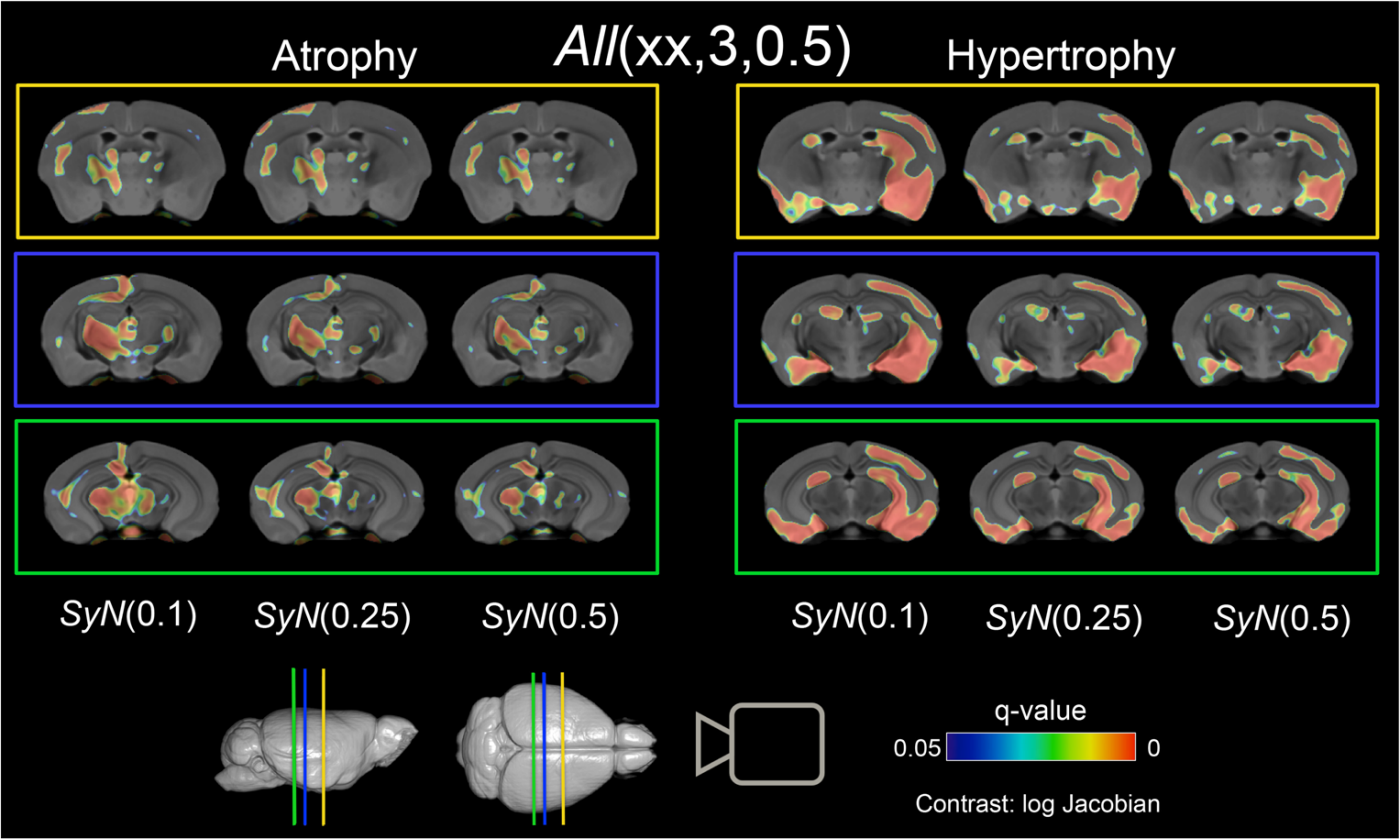
**The impact of SyN on the kainic acid VBA results, illustrated by corrected q-maps.** The parameter group *A*(xx,3,0.5) demonstrated notable effects when varying the SyN parameter. The number of significant voxels detected was highest using SyN(0.1). Little difference was found between SyN(0.25) and SyN(0.5) with the exception of a small region of atrophy in the ipsilateral hippocampus and adjacent cortex

Table 1 also indicates that using SyN(0.1) over SyN(0.25) results in large effect sizes, motivating us to examine the SyN effect for KA VBA (Fig. 7). We chose parameter group *A*(xx,3,0.5) since it featured large differences between the three SyN values across all the phantom metrics (see arrows in Supplemental Fig. S2). There was little difference in the atrophy detected near the caudate putamen (yellow slice, left). However, the extent and localization of the atrophy in the other slices varied with SyN. SyN(0.1) identified larger clusters in the cortex/corpus callosum and periventricular regions.

Volume increases (hypertrophy) in KA treated animals were apparent in the ipsilateral hemisphere, as well as in the contralateral amygdala and the adjacent hippocampus. Moreover, the contralateral caudate putamen showed hypertrophy. In general, the clusters extent increased as SyN decreased, which is consistent with parameters recommended for *antsRegistration* in human data (Tustison 2013). Similar comparisons of the VBA effects of RegU and MDT group can be found in **Supplemental Figs.****S4** and **S6**, respectively.

### Control Vs. Kainic Acid VBA

**Fig. 8:**
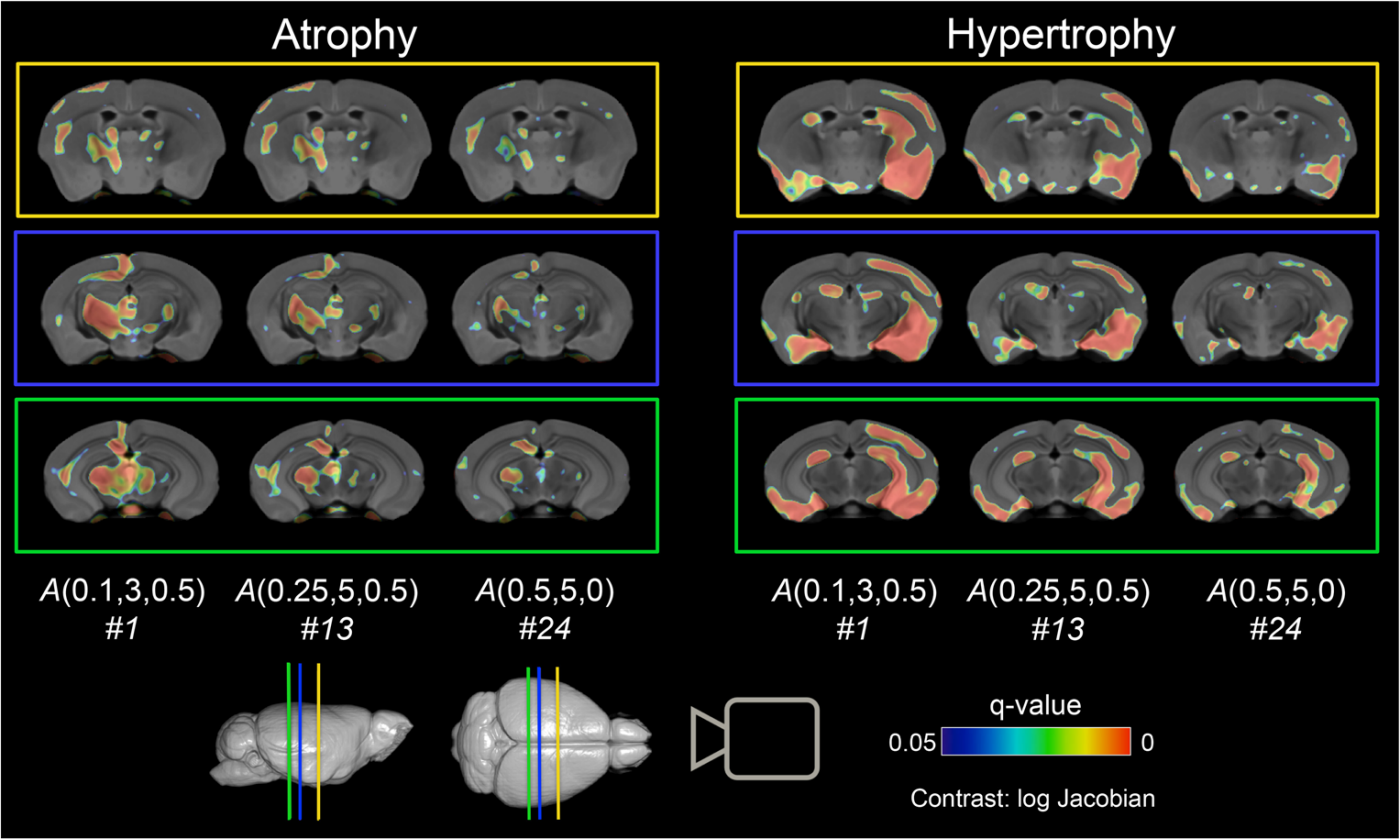
**Comparison of the kainic acid VBA results for the best, median, and poorest performing parameter groups according to the phantom metrics reveals the wide range of potential VBA results.** From left to right, the KA VBA results for the highest (*A*(0.1,3,0.5)), median (*A*(0.25,5,0.5)), and lowest (*A*(0.5,5,0)) rankings of the phantom metrics. This illustrates the variance within the typical parameter space, thus selecting an appropriate set of parameters is critical

Table 2 ranks all parameter groups according to the 4 phantom metrics. In general, the smaller SyNs, and more total regularization performed better, with *A*(0.1,3,0.5) as the top performer. For a comparison of the extreme and average cases according to the phantom rankings, the KA VBA results for this parameter set were plotted in Fig. 8 alongside the median case of *A*(0.25,5,0.5), and the worst performer *A*(0.5,5,0).

**Table 2:** The 4 phantom metrics of VBA pipeline evaluation, relative rank (italics), and average phantom rank (bold)

Parameter group	|Δd| (%)				*d’*				*(AUC–0.98)×100*				*TPR@p = 0.05(%)*				Phantom average	
	*Hc*		*CPu*		*Hc*		*CPu*		*Hc*		*CPu*		*Hc*		*CPu*			
C(0.1,3,0)	1.89	*11*	2.58	*19*	1.34	*10*	1.06	*6*	1.542	*12*	1.035	*12*	80.9	*10*	55.5	*11*	11.4	***12***
C(0.1,3,0.5)	2.41	*23*	2.89	*24*	1.64	*2*	1.38	*1*	1.645	*3*	1.572	*2*	92.6	*2*	97.7	*1*	7.3	***3***
C(0.1,5,0)	1.74	*4*	2.30	*6*	1.21	*19*	0.90	*17*	1.404	*18*	0.495	*17*	65.9	*15*	30.5	*16*	14.0	***15***
C(0.1,5,0.5)	2.08	*19*	2.54	*18*	1.47	*4*	1.20	*3*	1.633	*4*	1.395	*4*	90.0	*3*	92.9	*3*	7.3	***4***
C(0.25,3,0)	1.79	*7*	2.41	*9*	1.23	*16*	0.91	*16*	1.377	*19*	0.516	*16*	64.0	*17*	30.6	*15*	14.4	***16***
C(0.25,3,0.5)	1.90	*13*	2.49	*16*	1.38	*7*	1.08	*5*	1.593	*6*	1.248	*5*	85.8	*5*	76.4	*5*	7.8	***5***
C(0.25,5,0)	1.79	*9*	2.13	*3*	1.16	*24*	0.88	*21*	1.264	*23*	0.185	*22*	55.5	*22*	24.6	*21*	18.1	***21***
C(0.25,5,0.5)	1.76	*5*	2.43	*11*	1.31	*12*	1.01	*10*	1.544	*11*	1.097	*8*	81.2	*9*	60.0	*9*	9.4	***8***
C(0.5,3,0)	1.62	*2*	2.65	*21*	1.22	*17*	0.92	*15*	1.347	*20*	0.528	*15*	61.9	*18*	26.5	*19*	15.9	***18***
C(0.5,3,0.5)	1.77	*6*	2.66	*22*	1.37	*8*	1.03	*9*	1.562	*8*	1.133	*7*	83.5	*7*	63.8	*7*	9.3	***7***
C(0.5,5,0)	1.59	*1*	2.48	*13*	1.17	*23*	0.84	*22*	1.255	*24*	0.258	*20*	56.0	*21*	22.3	*22*	18.3	***22***
C(0.5,5,0.5)	1.68	*3*	2.42	*10*	1.30	*14*	0.99	*12*	1.528	*14*	1.054	*9*	79.5	*12*	55.9	*10*	10.5	***9***
A(0.1,3,0)	2.11	*20*	2.34	*7*	1.35	*9*	1.04	*8*	1.544	*10*	1.037	*11*	78.7	*13*	54.9	*12*	11.3	***11***
A(0.1,3,0.5)	2.45	*24*	2.86	*23*	1.66	*1*	1.35	*2*	1.662	*1*	1.617	*1*	92.8	*1*	97.6	*2*	6.9	***1***
A(0.1,5,0)	1.99	*15*	2.07	*2*	1.20	*20*	0.89	*20*	1.428	*15*	0.424	*18*	64.6	*16*	27.1	*18*	15.5	***17***
A(0.1,5,0.5)	2.13	*21*	2.48	*15*	1.49	*3*	1.16	*4*	1.652	*2*	1.409	*3*	89.9	*4*	92.1	*4*	7.0	***2***
A(0.25,3,0)	2.15	*22*	2.00	*1*	1.21	*18*	0.90	*18*	1.418	*16*	0.299	*19*	61.1	*19*	29.5	*17*	16.3	***19***
A(0.25,3,0.5)	2.03	*17*	2.48	*14*	1.44	*5*	1.04	*7*	1.615	*5*	1.202	*6*	85.0	*6*	73.4	*6*	8.3	***6***
A(0.25,5,0)	1.90	*12*	2.35	*8*	1.17	*21*	0.83	*24*	1.338	*21*	0.143	*23*	55.2	*23*	20.0	*24*	19.5	***23***
A(0.25,5,0.5)	1.87	*10*	2.44	*12*	1.34	*11*	0.97	*13*	1.557	*9*	1.029	*13*	80.3	*11*	54.4	*13*	11.5	***13***
A(0.5,3,0)	2.06	*18*	2.18	*5*	1.25	*15*	0.90	*19*	1.417	*17*	0.238	*21*	57.1	*20*	26.3	*20*	16.9	***20***
A(0.5,3,0.5)	1.92	*14*	2.62	*20*	1.39	*6*	0.99	*11*	1.570	*7*	1.052	*10*	82.1	*8*	60.0	*8*	10.5	***10***
A(0.5,5,0)	2.00	*16*	2.13	*4*	1.17	*22*	0.84	*23*	1.295	*22*	0.008	*24*	52.9	*24*	21.2	*23*	19.8	***24***
A(0.5,5,0.5)	1.79	*8*	2.51	*17*	1.30	*13*	0.93	*14*	1.529	*13*	0.926	*14*	77.2	*14*	46.8	*14*	13.4	***14***

*C*, only control group used for MDT; *A*, all individuals used for MDT, with (SyN, RegU, RegT)

Table 3 contains the results of the phantom rankings with the Dice coefficients, pipeline runtimes, the average phantom ranking from Table 2, and a ranked average of the three. This combined ranking favored larger steps sizes and smaller MDT groups due to faster convergence, and ranked *C*(0.25,5,0.5) as the top performer. Among the MDT(All) groups, *A*(0.1,5,0.5) was the top, and *A*(0.1,3,0) was the median performer.

**Table 3:** Dice, runtimes, average phantom rankings, and their combined average rankings, for the 24 parameter sets

Parameter group	*Dice % - 90%*				*Average*		*Runtime*		*Phantom Ranking*	*Dice + Runtime + Phantom Average*	
	*Hc*		*CPu*		*Dice*		*(Hours)*				
C(0.1,3,0)	2.66	*12*	3.23	*10*	11	*11*	85.0	*19*	*12*	14.0	*13 (tie)*
C(0.1,3,0.5)	2.51	*17*	2.99	*14*	15.5	*16*	134.0	*23*	*3*	14.0	*13 (tie)*
C(0.1,5,0)	2.37	*20*	2.95	*16*	18	*17*	33.2	*15*	*15*	15.7	*19*
C(0.1,5,0.5)	2.75	*10*	3.37	*5*	7.5	*7*	128.5	*22*	*4*	11.0	*10*
C(0.25,3,0)	1.26	*22*	2.48	*20*	21	*22*	11.2	*6*	*16*	14.7	*16*
C(0.25,3,0.5)	2.71	*11*	3.44	*2*	6.5	*5*	54.3	*17*	*5*	9.0	*7 (tie)*
C(0.25,5,0)	1.24	*23*	2.49	*18*	20.5	*21*	12.4	*9*	*21*	17.0	*22*
C(0.25,5,0.5)	2.76	*9*	3.41	*4*	6.5	*6*	8.6	*3*	*8*	5.7	*1*
C(0.5,3,0)	0.20	*24*	2.29	*24*	24	*24*	9.9	*4*	*18*	15.3	*17 (tie)*
C(0.5,3,0.5)	2.56	*14*	3.27	*8*	11	*12*	10.0	*5*	*7*	8.0	*2 (tie)*
C(0.5,5,0)	1.41	*21*	2.48	*19*	20	*20*	7.3	*1*	*22*	14.3	*15*
C(0.5,5,0.5)	2.51	*16*	3.20	*11*	13.5	*14*	7.5	*2*	*9*	8.3	*4 (tie)*
A(0.1,3,0)	3.26	*7*	3.25	*9*	8	*8*	111.5	*21*	*11*	13.3	*12*
A(0.1,3,0.5)	3.34	*5*	3.13	*13*	9	*9*	187.2	*24*	*1*	11.3	*11*
A(0.1,5,0)	3.18	*8*	2.98	*15*	11.5	*13*	48.5	*16*	*17*	15.3	*17 (tie)*
A(0.1,5,0.5)	3.58	*1*	3.43	*3*	2	*2*	90.5	*20*	*2*	8.0	*2 (tie)*
A(0.25,3,0)	2.59	*13*	2.59	*17*	15	*15*	19.1	*14*	*19*	16.0	*20*
A(0.25,3,0.5)	3.51	*2*	3.48	*1*	1.5	*1*	69.6	*18*	*6*	8.3	*4 (tie)*
A(0.25,5,0)	2.53	*15*	2.46	*22*	18.5	*18*	18.0	*13*	*23*	18.0	*23*
A(0.25,5,0.5)	3.40	*3*	3.34	*6*	4.5	*3*	13.7	*11*	*13*	9.0	*7 (tie)*
A(0.5,3,0)	2.40	*18*	2.47	*21*	19.5	*19*	13.5	*10*	*20*	16.3	*21*
A(0.5,3,0.5)	3.35	*4*	3.32	*7*	5.5	*4*	14.8	*12*	*10*	8.7	*6*
A(0.5,5,0)	2.39	*19*	2.37	*23*	21	*23*	12.0	*8*	*24*	18.3	*24*
A(0.5,5,0.5)	3.33	*6*	3.16	*12*	9	*10*	11.5	*7*	*14*	10.3	*9*

Informed by phantom studies, we examined the range of variations in VBA results for the KA-injected mice (Fig. 8). Results have been ordered from left to right according to their phantom rankings, the best on the left. It is apparent that VBA is highly sensitive to non-linear registration parameters. The significant contralateral regions of hypertrophy covered fewer and smaller areas with poorer registration performance, and there were hardly any significant voxels in the hippocampus and amygdala for *A*(0.5,5,0). The median parameter set detected much of the hypertrophy, but not to the extent of that of *A*(0.1,3,0.5), and largely missed the cluster in the caudate putamen. This can be summarized as a consistent increase of the number of false negatives and underreporting of treatment effects concurrent with poorer performance as reported by the metrics.

The atrophy observed for the best performer was mostly ipsilateral to the injection site, localized to the hippocampus, hypothalamus, cingulate cortex, primary somatosensory and temporal association cortex, as well as the caudate putamen, globus pallidus and thalamus (anterodorsal, ventral, reticular nuclei). Contralateral atrophy was also noted, to a smaller extent, in the medial geniculate, hypothalamus, temporal association cortex, and the periventricular hippocampus. Approximately a third of these regions would not be reported as significant based on the median parameter set, and another third would be overlooked by the poorest performer. In both scenarios, the choice of SyN parameters had a considerable impact on the VBA conclusions.

## Discussion and Conclusions

Phenotyping rodent models of neurological and psychiatric conditions poses substantial challenges because of the number and size of the images to be analyzed. Image analysis pipelines aim to provide quantitative image-based biomarkers, in a reproducible and automated manner, while meeting the needs for accuracy and efficiency. Previous efforts have been largely dedicated to automating pipelines for human brain images, and several efforts have been made for rodent brain images (Ad-Dab’bagh et al. 2006; Sawiak et al. 2009a). Existing methods for evaluating VBA only capture aspects of the processing chain. Here, we present SAMBA, a VBA pipeline for the rodent brain with an HPC implementation, and an unprecedented extensive validation effort. HPC resources were used to produce 24 variations of VBA in a mouse model of epilepsy, to identify the most reliable results. Our validation framework is based on simulated atrophy/hypertrophy phantoms. Combined, our work enables timely preclinical VBA with increased confidence.

### Comparison to Previous Work

Pagani et al. (2016) described a VBA pipeline for rodent brain MRI, which relies on ANTs, and features segmentation, label-based analysis, cortical thickness, and VBA. Our approach handles larger and multivariate image sets, using 7 derived DTI contrasts. Because our images have almost 2 times higher resolution, our typical arrays of 512x256x256 voxels are 6 times larger. To meet the computational demands of high-resolution image analysis we designed our pipeline for an HPC environment. A defining feature of our pipeline is that it provides the needed code infrastructure to run in an HPC environment.

A second distinctive feature is the proposed validation framework. To the best of our knowledge, a complete validation framework does not exist for preclinical VBA. However, several aspects of the current framework have historical foundations. Freeborough and Fox (1997) used the Boundary Shift Integral to simulate volumetric change. To achieve more anatomically realistic morphological changes Camara et al. (2006) used physical tissue models in conjunction with finite-element analysis; while Karaçali and Davatzikos (2006) preserved topology by constraining the Jacobians of the deformation fields. These methods are best suited for higher-level mammalian brains that feature extensive folding, which interfaces directly with cerebral-spinal fluid (CSF). For the mouse, VanEede et al. (2013) used Jacobian regularization to simulate both atrophy and hypertrophy. While this resulted in Jacobians that were more uniform and spatially constrained, ours has the advantage of requiring substantially less computation time.

A potential limitation of our work is that the volume changes induced in our phantoms (~14%) may not have been enough to emulate the large deformations in the KA study. On the other hand, it may be more difficult to recover changes of ~10% or less. A future task is to establish a method to quickly produce custom volume changes.

A key component of the validation framework comes from the evaluation metrics. Most often the accuracy for spatial normalization is quantified by label overlap metrics such as Dice or Jaccard coefficients (Avants et al. 2011), or label “entropy” based on lower order tissue segmentations (Robbins et al. 2004). However, these do not fully capture the entire VBA process. More appropriate for VBA, Shen et al. (2007) looked at the number of voxels in which they had induced atrophy, and measured the difference between this target and the number of significant atrophy voxels recovered. Similarly VanEede et al. (2013) compared simulated and recovered atrophy/hypertrophy via Deformation Based Analysis (DBA), to measure the number of true and false positives. While the latter two served as inspiration for some of the metrics we employed, we provide multiple quantitative metrics, and note that our absolute distance to target is based on the effect size, as inherited from SurfStat VBA (Worsley et al. 2009), which is not normalized by the standard deviation.

The sensitivity index has similarities to past work, e.g. VanEede et al. (2013) showed that by excluding the significant voxels in an *r* = 3 voxel shell surrounding a regions of interest, one could eliminate most false positives. This shell is in principle equivalent to our leakage region. Rather than omitting these “almost-correct” voxels, we have used the effect sizes to quantify the precision of spatial normalization and the effect of the smoothing.

### Temporal Performance

While running parallel jobs across multiple nodes reduces computation time, the need for wise resource management remains. In the simplest case, each job is distributed to one node, which is not efficient (Fig. 4). In our preclinical studies requiring 10–25 concurrent jobs, 3 nodes provided a good balance between runtime and efficient resource management. Surprisingly, using 4 nodes can take *longer* than using 3 (Fig. 4a) if two or more particularly demanding jobs were assigned to the same node. Distributing such jobs to even out resource demands can significantly improve efficiency. Figure 4c, d show large discrepancies in runtimes for smaller SyN values, indicating that some registration jobs converged slowly, or suffered from oscillations with little improvement in quality. To circumvent this, one can limit the iterations at the fully sampled level to ~60 or less. Such strategies can reduce VBA runtime significantly—and are the subject for future work.

In principle, computational expenses are relatively cheap compared to the cost for producing animal models and the imaging equipment acquisition, maintenance and operation. In practice, analysis often takes the longest amount of time in an experiment. With our efforts, we try to balance the computational time required to reach a conclusion at the end of an experiment.

### Dice Coefficients and Phantom VBA Metrics

Due to the computational efficiency of the HPC implementation, we were able to explore the parameter space of the non-linear registration processes. This provided insight into the value of phantom metrics, and the importance of registration/MDT choices. Dice coefficients captured the benefit of using the All MDT groups, which the phantom metrics largely failed to do. However, apart from detecting the impact of using RegT(0.5), the Dice did not provide much direction on which registration parameters to use.

|The metric Δ*d*| had a non-linear relationship with the choice of parameters, and did not detect significant differences in either the atrophic or hypertrophic cases. In general, the best aggregate performing parameters saw poor performance according to this metric. This is due in part to the demand for capturing a spectrum of diffeomorphic changes with a single set of registration parameters, and the various forms of smoothing occurring throughout the pipeline. A |Δ*d*| penalty in accuracy was incurred for improved spatial and statistical sensitivity (*d’* and ROC metrics, respectively). While it is not recommended to use |Δ*d*| for optimization, it remains a vital piece of information to be shared alongside VBA results, as it estimates the error in effect sizes, particularly if the magnitude of an effect size is critical to one’s study.

In contrast to |Δ*d*|, *d’* was sensitive to virtually any change in the parameters. Assuming that spatial localization is given priority over effect size, it is appropriate to include *d’* in any VBA tuning/optimization. This provides an idea of the uncertainty associated with the spatial extent of observed effects. Further, it can characterize the “VBA SNR” of the system. Note that *d’* depends on the spatial smoothing kernel, an aspect which we did not explore.

The ROC metrics were sensitive to parameter changes, even more than *d’*, and typically had the smallest *p* values. They were more sensitive to hypertrophy, as their variance was considerably wider here than in the atrophic scenarios. Like *d’*, these metrics are appropriate candidates for VBA tuning. Beyond adjusting the processing parameters, the ROC provides motivation for a particular *p-* or *q*- threshold. Using this to estimate the TPR and FPR in the real data, one can choose where on the curve to report results, depending on which type of Error (Type 1 vs. Type 2) is more tolerable. Including phantom ROCs should increase the level of transparency and confidence in preclinical VBA results, and will hopefully contribute to wider-spread adoption.

While Dice coefficients are an established standard, they are obtained through labor-intensive manual editing, susceptible to bias, and impractical for routine use. Of the phantom metrics, the best candidate for a Dice substitute was the AUC, the two having a high correlation. However, comparing MDT(Controls) and MDT(All) revealed an important shortcoming of using phantoms, i.e. the dependence on the induced volume change. We note that the ~14% volume change was substantially less than what we encountered in the KA data, and it is critical to include this when reporting phantom metrics. It is possible that other metrics would correlate strongly with Dice, had larger volumetric changes been induced in the phantoms, or smaller structures been chosen for analysis. Future phantoms can be tuned to better simulate the data in question, and would require a more sophisticated model beyond the linear expansion/contraction method used here.

### Selecting Registration and MDT Parameters

Apart from which MDT group to use, the phantom metrics gave clear insight into which registration parameter values are more likely to give high quality results: SyN(0.1), RegU(3), and RegT(0.5). As a general application, the phantom metrics could aid in selecting between a limited number of parameters. For example, one may already be confident that SyN(0.3) balances quality of results and runtime, but may want to tune RegT to find the value predicted by ROC metrics to provide the highest TPR/FPR ratio. Sharing such tuning procedures and the relevant phantom metrics will help build the experience of the community. Currently, it seems difficult to find detailed registration parameters reported, much less a justification for their choice and the implications for interpretation of VBA results.

The effect sizes of each parameter on the performance metrics and runtimes can inform the decision on how to get reliable results in a reasonable timeframe. The rankings of Table 2 are a step towards incorporating such results into a cost-benefit analysis. Instead of simply choosing the highest ranked parameter group according to the phantom metrics (and Dice, if available), it may be wise to take into account that even though *A*(0.1,3,0.5) promises the results with the highest fidelity, it also required the longest time (~1 week). By weighting the Dice and phantom metrics against the runtime, one can get a more balanced sense of “value”, particularly if access to high-powered computing resources is limited. Such a weighted ranking is included in Table 3, and indicates that *C*(0.25,5,0.5) can deliver results in the upper third of quality, in under 9 h. Another strategy might be to recognize a mismatch between the expected effect size and runtimes. An obvious example here is RegT(0.5), which provides benefits in quality that make it worth the ~1/3 increase in time. Although the phantom metrics did not elucidate the benefit of using all subjects to construct the MDT observed in the KA VBA results, the pairwise temporal analysis revealed that one can expect it to take 50% longer—and that such a sacrifice is a low (and predictable) price to pay for the benefit of a minimally-biased template. Taking these last two points into consideration, when notable deformations are expected we would recommend default parameters of *A*(0.25,5,0.5) (ranked 7th) for expedited results or *A*(0.25,3,0.5) (ranked 4th) if one can afford the modest increase in processing time. For more subtle deformations, a more appropriate range for RegU might be 1–3 voxels, but will take longer. If tuning the parameters, we recommend the stable range of 0.3–0.7 for RegT. While SyN(0.1) tended to produce the highest quality results, it should be reserved for cases when avoiding false negatives is critical and the processing time and/or HPC resources can be spared.

Voxel-based Analysis is overall a complex process. It is expected that optimal parameters will vary based on several other key factors not addressed here, such as the contrast(s) used for skull-stripping and registration, array size, voxel size, and biological variations across a given study. While parameter recommendations can be of value, it is important to remember “one size does NOT always fit all.” Therein lies the key strengths of this work: a toolbox for efficiently performing preclinical VBA and a framework for evaluating the quality of the results. Combined with the open-source nature of the code, the latter of these makes it easier for a researcher to identify parameters that are appropriate for their data.

### Kainic Acid VBA

We detected atrophy in the amygdala near the injection site and the hippocampus, and also in the striatum and thalamic nuclei (e.g. the geniculate bodies, zone incerta, and laterodorsal nucleus). Changes in the ipsilateral hippocampus, striatum, pallidum and thalamus have been well documented in patients with temporal lobe epilepsy (Dreifuss et al. 2001). This study also reported contralateral atrophy in these structures. Of these we detected atrophy in the contralateral thalamus and periventricular hippocampus. We also detected widespread contralateral hypertrophy. There is evidence of contralateral hypertrophy in rodent brains under similar circumstances (Pearson et al. 1986; Dedeurwaerdere et al. 2012). These can be explained by hippocampal neurogenesis (Parent et al. 1997), mossy fiber sprouting (Wuarin and Dudek 1996), astrogliosis (Li et al. 2008)–—and could obscure the VBA detection of atrophy due to neuronal cell death (Altar and Baudry 1990).

**Fig. 9:**
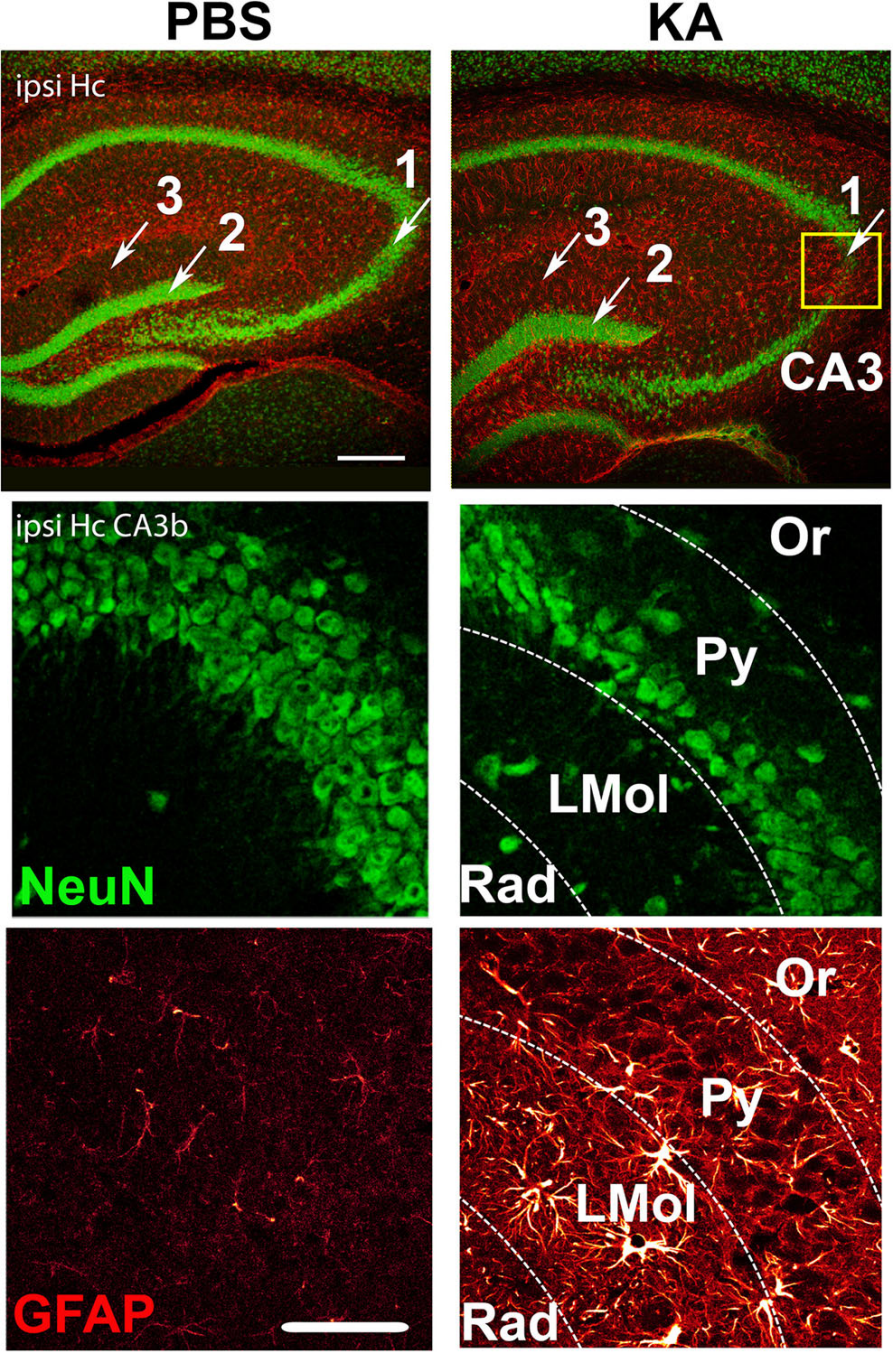
**Histology of the ipsilateral hippocampus using NeuN and GFAP immunoreactivity revealed that KA injected animals present concurrent pathologies**. The yellow box represents the CA3 hippocampal area, enlarged in the two lower rows. The arrows show: 1) neurodegeneration in the pyramidal cell layers, in the CA1 and in particular CA3 areas (scale bar, 200 μm); 2) granule cells dispersion; 3) astrogliosis (scale bar, 20 μm). Abbreviations: Or-stratum oriens, Py-pyramidal layer, LMol-lacunosum moleculare, Rad-stratum radiatum, ipsiHc ipsilateral hippocampus

To validate VBA results with histology (Fig. 9), we examined the hippocampus of a KA injected mouse and a PBS injected control. Neurons and astrocytes were visualized using a Leica TCS SL confocal microscope, after staining with antibodies against neuronal nuclei (NeuN, Millipore) and glial fibrillary acidic protein (GFAP, Sigma). The histology revealed neurodegeneration and astrogliosis in KA injected animals. Arrow 1 highlights neurodegeneration in the pyramidal cell layers, in the CA1 and in particular CA3 areas. The differences in the dispersion of the granule cells are indicated by Arrow 2, while Arrow 3 shows the region where astrogliosis is significant. Thus it is seen that the histology supports the VBM differences between KA injected and control animals.

The choice of registration parameters impacted the detection of brain phenotypes, highlighting the need for mindful VBA. This was evident when varying RegT, where we noted the potential for divergent interpretations. Similar variations in sensitivity may arise if working at a fixed statistical threshold, and if the dataset under consideration has similar variability to ours. One might select registration parameters based on intuition, and our “best-guess” of *C*(0.25,3,0.5) was not far from the median performer *A*(0.25,5,0.5). Figure 8 showed modest variations between *A*(0.25,5,0.5) and *A*(0.1,3,0.5) in the atrophic ipsilateral regions, particularly the hippocampus, and also the striatum, pallidum, cingulate cortex, thalamus and hypothalamus. There was however more variation in the contralateral hypertrophy in the amygdala, cortex, striatum, and hippocampus. Hypertrophy largely vanished when using poorer performing parameter sets e.g. *A*(0.5,5,0). This may be a compensatory mechanism for severe atrophy in one hemisphere, under the constraint that volume needs to be preserved when all brains are mapped into the same template space. However, we have not seen such compensatory behavior of contralateral hypertrophy in our synthetic studies. Therefore, while it is possible that severe atrophy poses substantial challenges for automated pipelines, we should also acknowledge that real biological effects such as swelling or astrogliosis might contribute to enlargement not just locally but also in contralateral brain areas.

The overall variability between registration parameter sets underscores the importance of a method for validating VBA, to protect against conforming the results or their interpretation to a preconceived bias. This translates into a need to develop quantitative tools for informing VBA, not only on registration parameters, but also on statistical thresholds and smoothing kernels (Jones et al. 2005). Such tools should allow decisions to be made using a consistent framework, imbuing confidence to researchers, and their audience. The phantoms and the evaluation methods we proposed are starting points for such a toolbox/framework.

### Future Work

We have applied the pipeline in its entirety, or as independent modules to phenotyping live (Badea et al. 2016), or fixed mouse (Badea et al. 2007, 2012), rat (Calabrese et al. 2013), and primate brain images (Calabrese et al. 2015a). Our future efforts are motivated by the desire for efficient and reliable voxel-based analysis, which addresses an unmet need for validating and sharing VBA results in preclinical MRI. To realize this vision, we need to identify the minimum quantitative validation requirements to become standard in future VBA studies. It would be beneficial to standardize workflows for generating data sets with a range of simulated atrophy and hypertrophy. There is a need for comprehensive, well-characterized evaluation metrics. Phantoms can guide the VBA processing and interpretation of real data. We should next extend the phantom concept beyond VBM, to other contrasts. We have optimized critical parameters and note that there is potential for more efficient algorithms. The effects of other parameters, e.g. the size of the smoothing kernel, need to be more thoroughly investigated. Future work might also consider validation models that employ biologically relevant deformations, a greater range of scales, landmark distances, or region-wise overlaps, as in (Tustison 2013). While we have incorporated options for both parametric and nonparametric statistics, we have only explored the first case here, and more can be done varying the options for statistical analyses. A deeper consideration of preclinical study design—from data collection to analysis strategy, and statistical modeling—is warranted due to the potential to improve inference from preclinical to clinical studies. Also, a fully determined BIDS standard for small animal imaging and derived data is still a work in progress.

In conclusion, it is clear that parallelizing tasks such as image registration and statistical analysis (in particular permutation based nonparametric tests) are worth the effort. Yet, this is not yet widely-adopted, in part because of the upfront effort required for such implementations. We shared our experience in the context of small animal brain image analysis, using a local computing cluster. Further developments should address portability to the cloud. While we focused on the brain, such efforts are translatable to other organs (such as heart and lungs), and other species (rats, non-human primates). Lastly, we argue that validation efforts have not received sufficient attention in preclinical VBA, and we propose an evaluation framework, also easily adaptable to other organs and species.

## Conclusions

We addressed the demands of preclinical VBA with an automated pipeline in a local HPC cluster. We identified a need for optimization and validation tools. To address this, we proposed several evaluation metrics to be used in conjunction with phantoms featuring simulated atrophy and hypertrophy. We applied these tools to illustrate how widely VBA results can vary with different registration parameters, using as an example a mouse model of epilepsy. Using such tools, we are able to increase the confidence in VBA results, and quantitatively communicate this confidence. The community shall benefit from further development of a robust evaluation framework for preclinical VBA studies, whether these are performed in local computing environments, university/company HPC resources, or in the cloud.

### Information Sharing Statement

The input DWi and FA data sets used in this work and the software code are freely available at https://github.com/andersonion/VBA_validation_framework (phantom generation, evaluation metrics); and https://github.com/andersonion/SAMBA (for the VBA pipeline).

## Electronic Supplementary Material

ESM 1 (PDF 26 kb)

ESM 2 (PDF 2387 kb)
