# Supplementary material for: Small Animal Multivariate Brain Analysis (SAMBA) – a High Throughput Pipeline with a Validation Framework
Source: Neuroinformatics. 2018 Dec 19;17(3):451–72. doi: 10.1007/s12021-018-9410-0 (PMC6584586; doi:10.1007/s12021-018-9410-0)
Supplement: Supplementary file 1 — (PDF 26 kb) [file 12021_2018_9410_MOESM1_ESM.pdf]

Parameter Comparison		*Left Hc*	*Right Hc*	*Left CPu*	*Right CPu*
**SyN:**	*p*-value:	*5.1e-05	*9.6e-04	0.054	0.013
0.1 > 0.25	effect size:	**0.32%	**0.40%	0.30%	0.24%
	p-value:	*9.3e-08	*3.3e-04	*0.003	*1.3e-04
0.1 > 0.5	effect size:	**0.51%	**0.55%	**0.42%	**0.37%
	p-value:	*2.2e-10	0.023	*1.5e-07	*6.0e-07
0.25 > 0.5	effect size:	**0.19%	0.21%	**0.09%	**0.12%
**RegU:**	*p*-value:	0.040	0.290	0.825	0.456
3 > 5	effect size:	0.11%	0.04%	0.05%	0.07%
**RegT:**	*p*-value:	*2.1e-14	*1.2e-08	*1.6e-11	*2.4-11
0.5 > 0	effect size:	**0.81%	**0.71%	**0.70%	**0.63%
**MDT:**	*p*-value:	0.524	*1.7e-08	*8.4e-12	0.518
All > Ctrl	effect size:	0.03%	**0.68%	**0.25%	0.09%

Table S1. Paired *t*-tests comparing Dice coefficients in the kainic acid group for different values of the 4 processing parameters. Substantial atrophy occurred in the Right Hc and is considered to be “treated,” while the Left CPu experienced minimal volumetric change and functions as a control. For SyN (0.1 > 0.25) and (0.1 > 0.5), mild but significant effect sizes were seen in most cases. For SyN (0.25 > 0.5) effect sizes were 2-3x smaller, but still significant. **p*-value < 0.01; **corresponding effect size
