# Supplementary material for: Small Animal Multivariate Brain Analysis (SAMBA) – a High Throughput Pipeline with a Validation Framework
Source: Neuroinformatics. 2018 Dec 19;17(3):451–72. doi: 10.1007/s12021-018-9410-0 (PMC6584586; doi:10.1007/s12021-018-9410-0)
Supplement: Supplementary file 2 — (PDF 2387 kb) [file 12021_2018_9410_MOESM2_ESM.pdf]

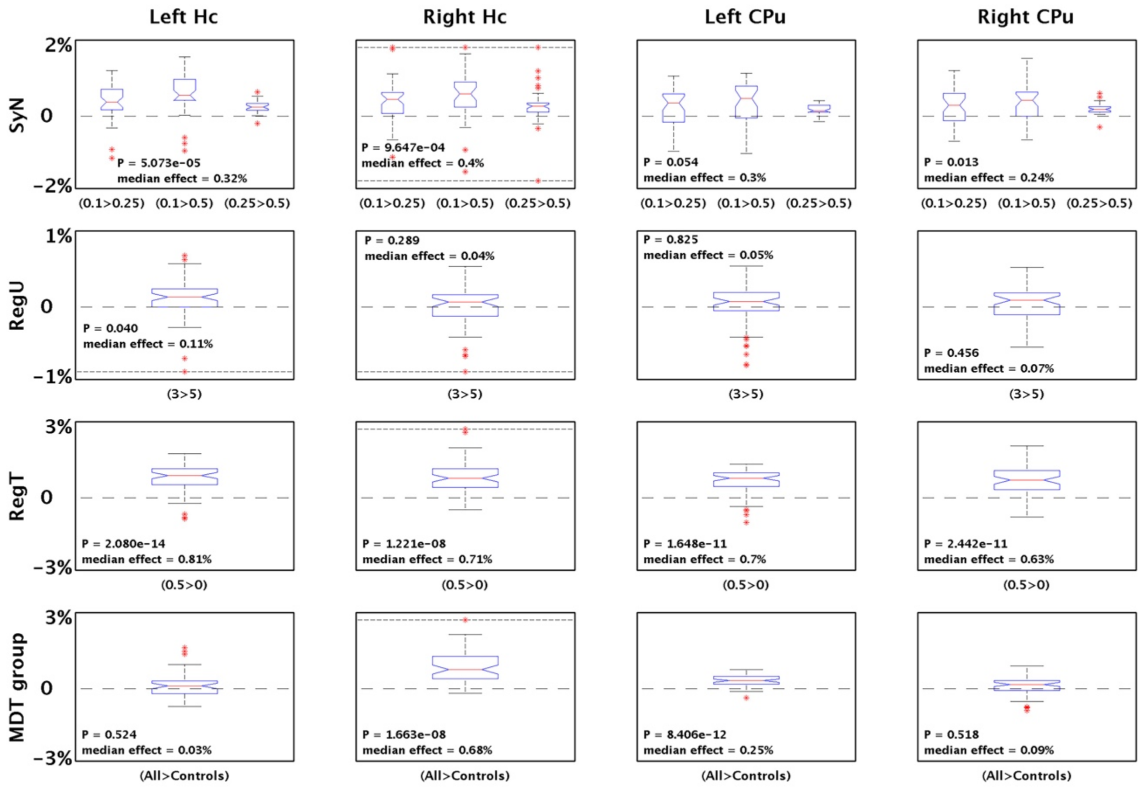

Fig. S1. Subject-wise paired *t*-test comparisons of differential changes in the Dice coefficient for 4 structures (columns) of the Kainic Acid mice, as the 4 key parameters are varied (rows). The insets of the SyN comparison show only the effect size and *p*-values for the (0.1 > 0.25) tests. Varying RegT had the strongest effect on the Dice coefficients, followed by SyN. No discernable differences were detected between RegU(3) and RegU(5) by the Dice. Notably, using the All MDT group was better for detecting the large atrophy in the Right Hc, without incurring a penalty in the other regions.

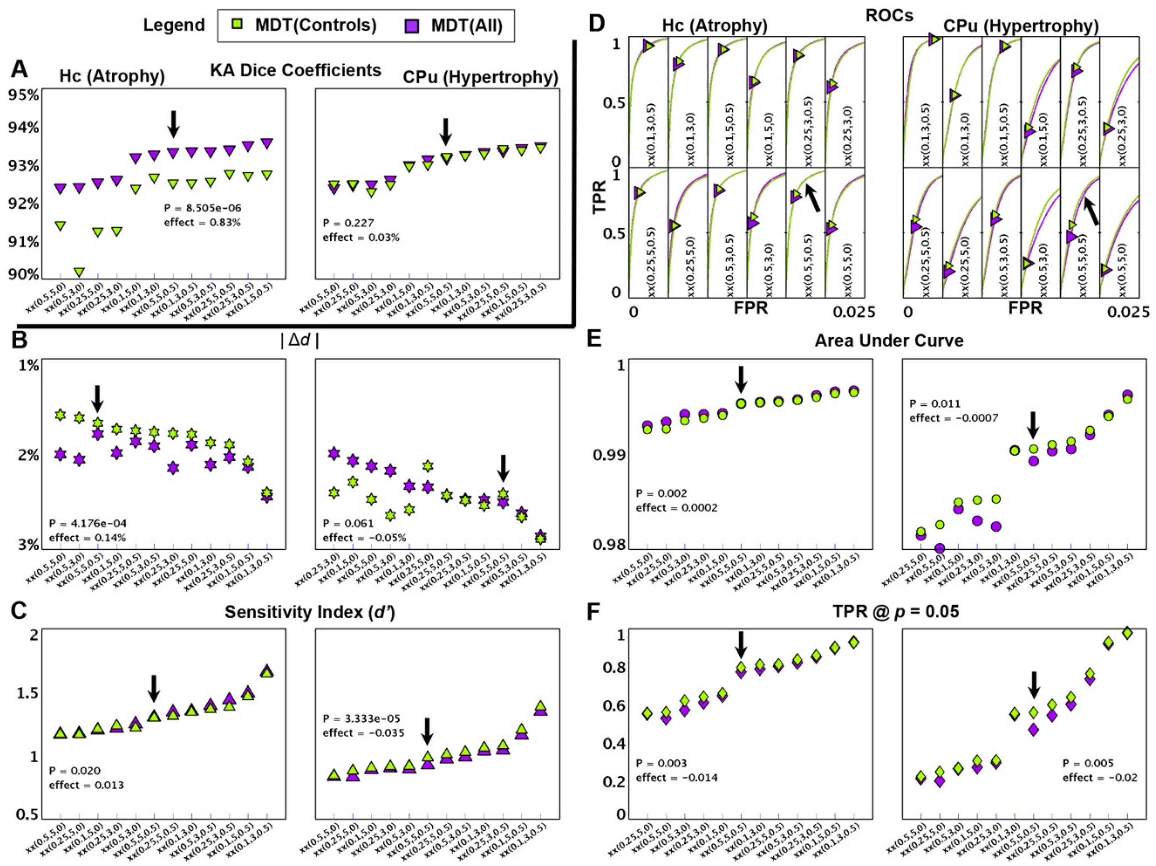

Fig. S2. Varying SyN had a modest effect on the various performance metrics. Closer inspection of the large Dice effects (A) indicated that RegT(0.5) equalized the performances of SyN(0.1) (green), SyN(0.25) (purple), and SyN(0.5) (red), while the consistent drops in Dice for SyN(0.25) and SyN(0.5) were due to RegT(0). With the exception of |Δ*d*| (B), SyN(0.1) had a positive impact on performance on all phantom metrics (C-F). In most cases, using 0.25 instead of 0.5 voxels made a minimal difference. The arrows point to parameter group *A*(xx,3,0.5), which was chosen for KA VBA comparison in Figure 7. Note that this choice shows large differences between the three SyN values across all the phantom metrics—but not Dice.

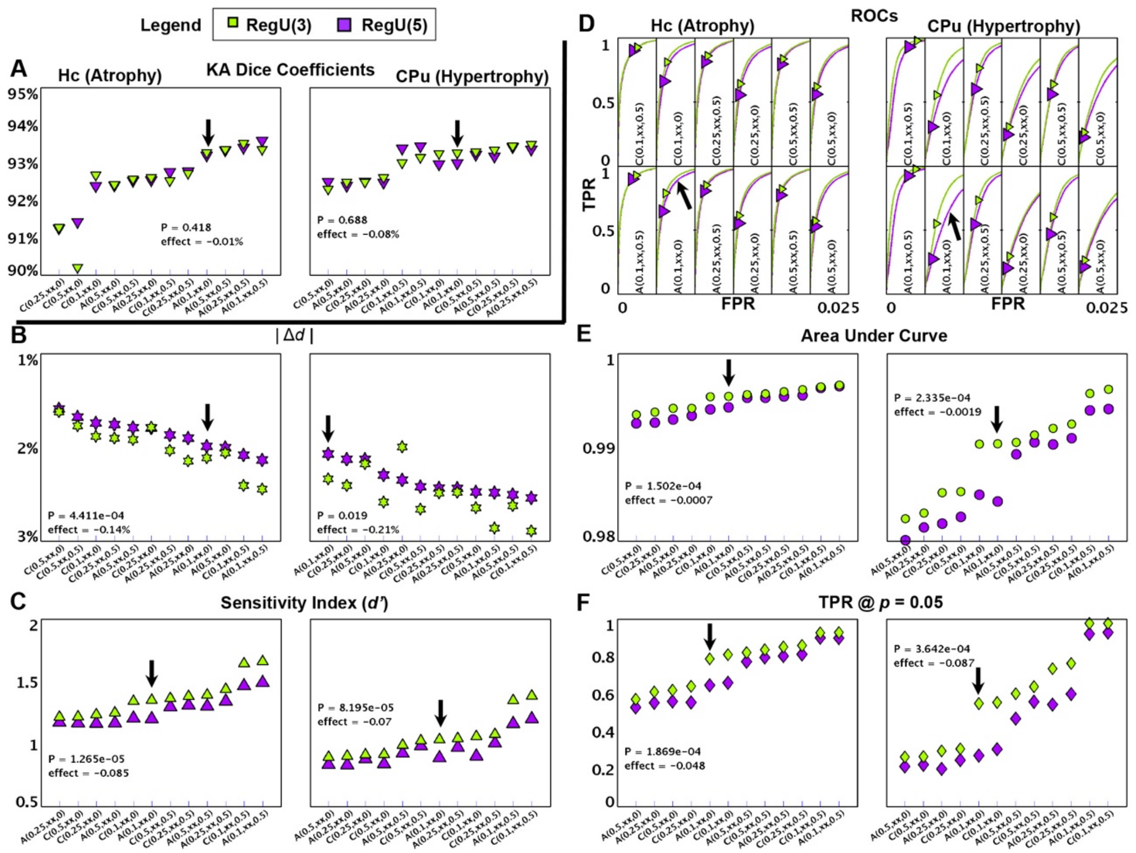

Fig. S3. The phantom metrics revealed slight effects when using RegU(3) over RegU(5). The traditional Dice coefficients (A) did not detect significant difference in performance between the two RegU values. In contrast, the phantom metrics (B-F) all noted a small, yet significant effect size in favor of using RegU(3). Effect sizes were ~2x smaller than those produced by varying SyN. The arrows indicate the group chosen for Figure S4, *A*(0.1,xx,0), because of its large differences in the hypertrophic AUC and TPR values (right panels of E & F).

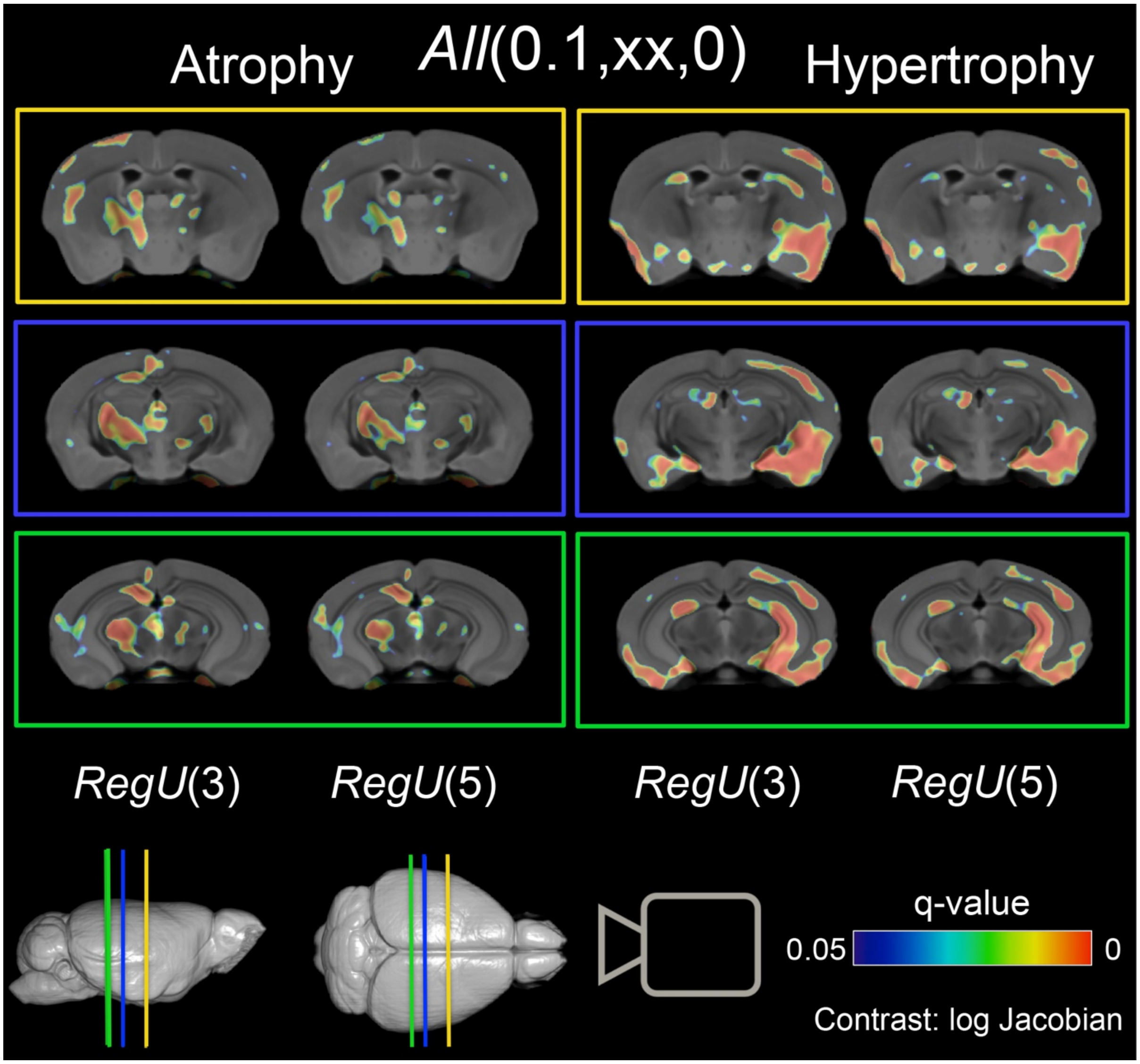

Fig. S4. Varying RegU at *A*(0.1,xx,0) produced slight changes in the kainic acid VBA results. The phantom metrics predicted small variations due to RegU in the KA VBA results of the parameter group *A*(0.1,xx,0). The extent of the significant voxels are consistent with this, with RegU(3) resulting in slightly larger clusters. This is evident in the atrophy in the periventricular regions, for example. Using RegU(5) greatly diminished the hypertrophy detected in the contralateral corpus callosum and cortex.

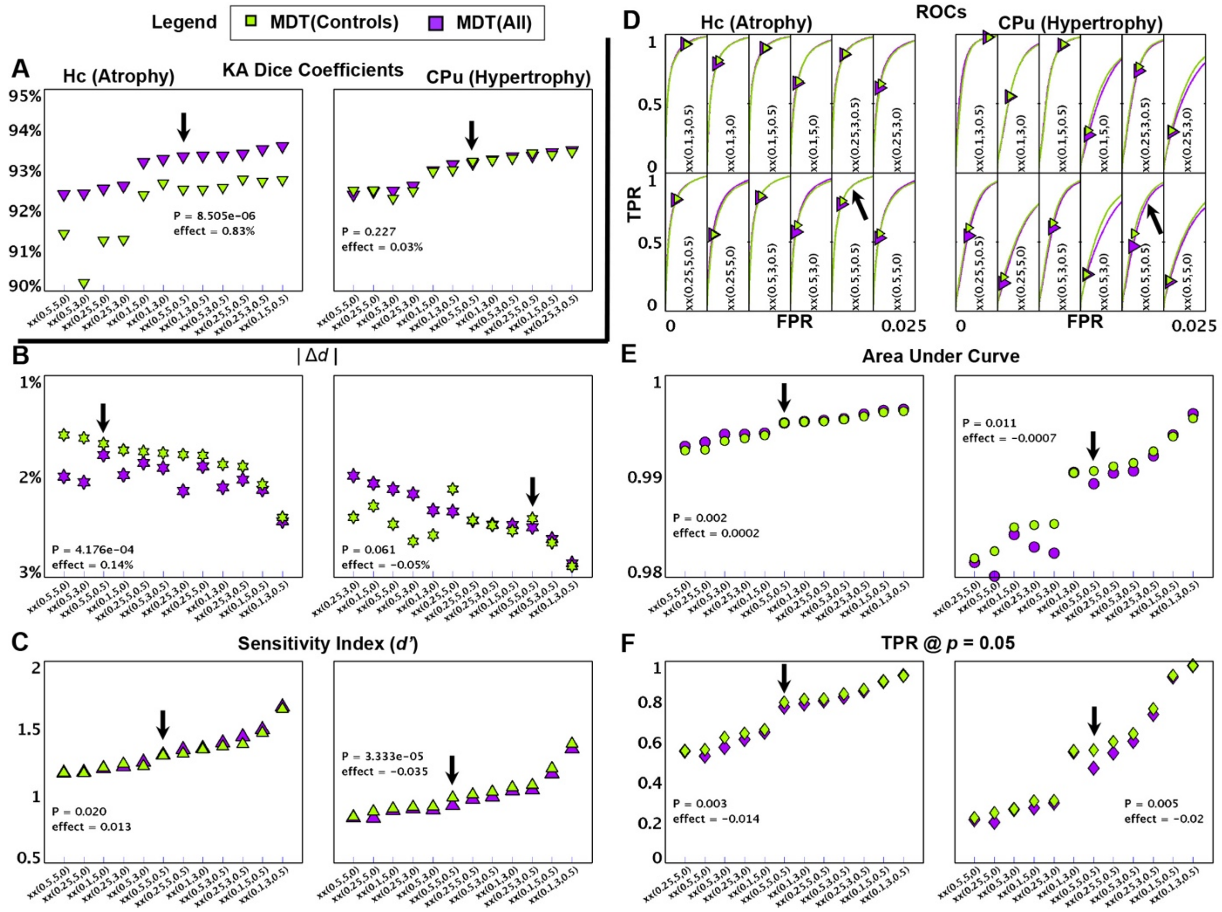

Fig. S5. Only the KA Dice coefficients reported a significant advantage of using All subjects to construct the MDT. MDT(All) greatly improved the Dice values (A) in the region of large deformations. The phantom metrics (B-F) appeared indifferent to the MDT group, indicating that a phantom with larger synthetic volumetric changes would likely result in better correlations between the phantom metrics and the performance of the real KA data. For KA VBA comparison in Figure S6, parameter group *xx*(0.5,5,0.5) (arrows) was selected to illustrate the effects only the Dice coefficients were able to capture.

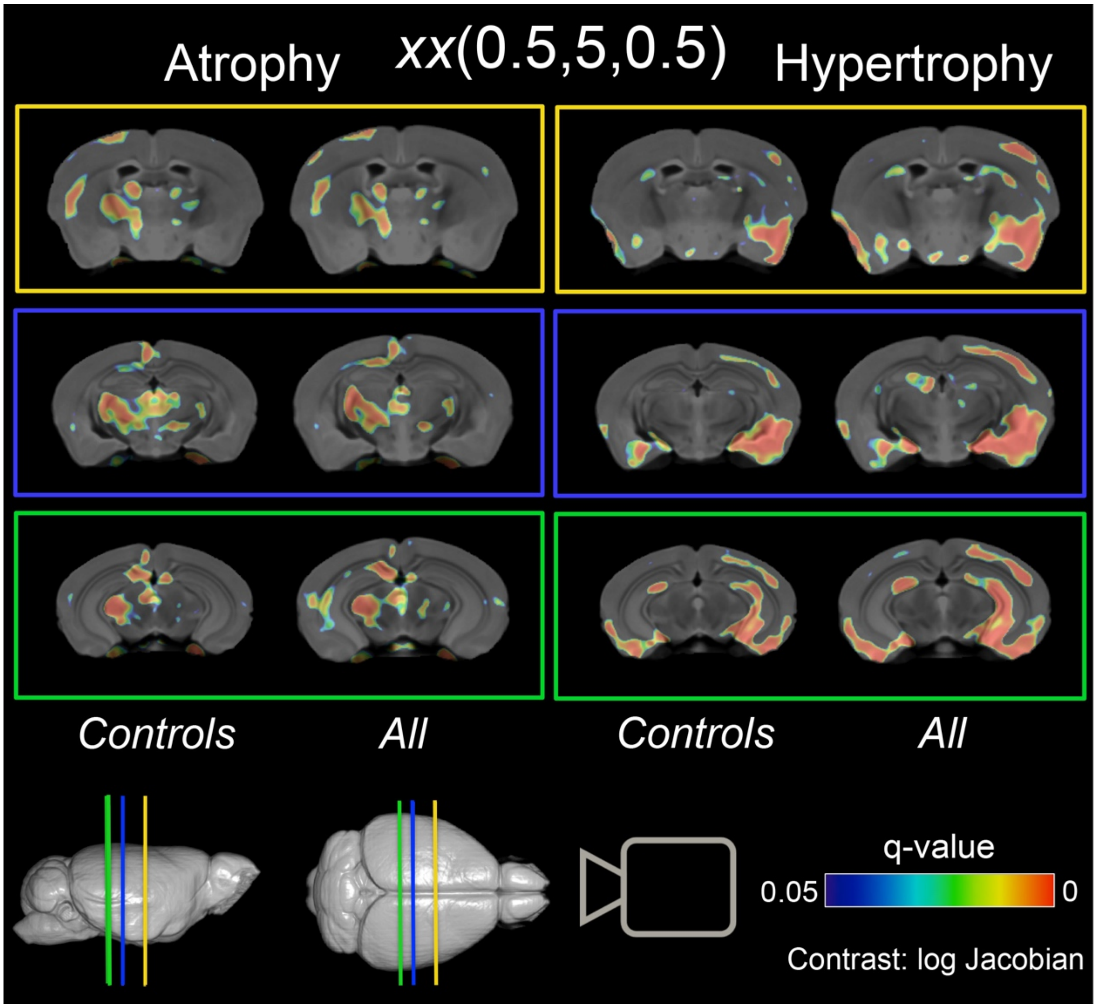

Fig. S6. Kainic acid VBA results for the two MDT groups at xx(0.5,5,0.5) showed that using All subjects had an effect on the kainic acid VBA results not indicated by the phantom metrics. Substantially more localized hippocampal atrophy was detected when using MDT(All). Unexpectedly, the largest gains in detection were in regions of hypertrophy contralateral to the injection site in the cortex, caudate putamen, amygdala, and hippocampus. MDT(All) detected ipsilateral hypertrophy near the midline and hippocampus, which otherwise would have been unreported. More atrophic affects were detected in the center of the brain when using MDT(Controls). These differences in the KA VBA results were expected based on the Dice coefficients, but were not indicated by the phantom metrics.

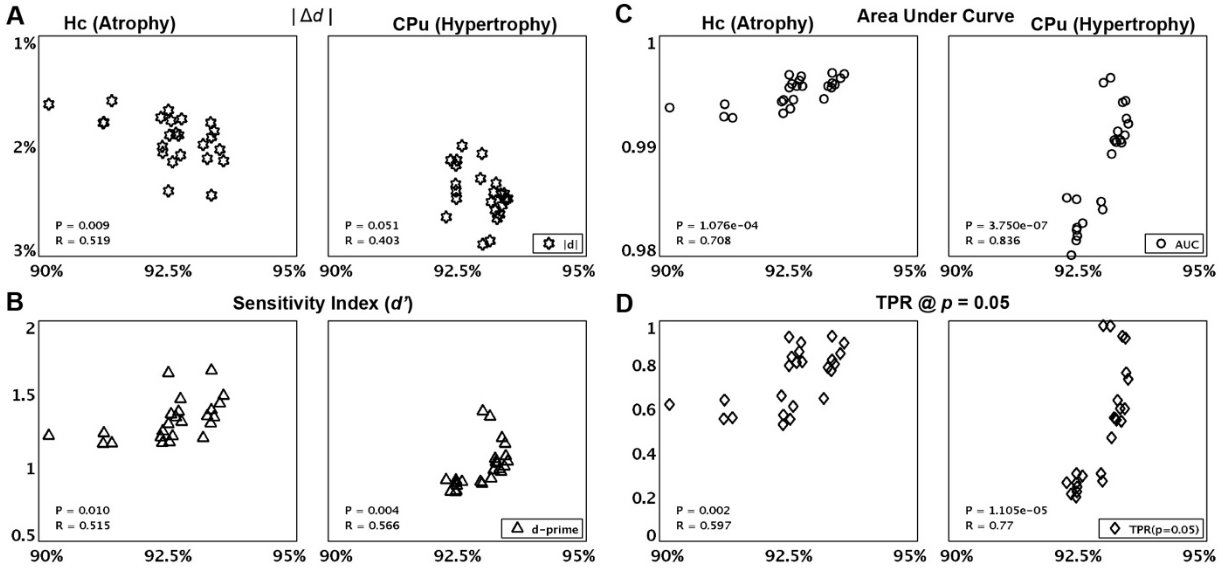

Fig. S7. Correlations between Dice coefficients and the phantom metrics |Δ*d*| (A), *d'* (B), AUC (C), and TPR @ *p* = 0.05 (D) are visualized in scatter plot form. While statistically significant ($p < 0.05$) correlations were observed between the phantom metrics and the Dice values, the relationships differed between regions of atrophy and hypertrophy, confounding any generalized relationship between the two. The large respective values of $R = 0.708$ and 0.836 for the AUC indicate that it is the leading phantom metric for predicting how the Dice coefficients might perform when they are otherwise unavailable.
